# Fetal endocrine axes mRNA expression levels are related to sex and intrauterine position

**DOI:** 10.1186/s13293-024-00637-9

**Published:** 2024-08-05

**Authors:** Ariel Yael, Ruth Fishman, Devorah Matas, Tirza Doniger, Yoni Vortman, Lee Koren

**Affiliations:** 1 The Faculty of Life Sciences, Bar-Ilan University, 5290002 Ramat Gan, Israel; 2 Department of Brain Sciences, Weizmann Institute of Science, 76100 Rehovot, Israel; 3 Department of Animal Sciences, Hula Research Center, Tel Hai Academic College, Upper Galilee, 1220800 Qiryat Shemona, Israel

**Keywords:** Cortisol, Glucocorticoids, Hair testing, HPA-axis, HPG-axis, Intrauterine position, *Myocastor coypus*, Sex differences, Steroid receptors, Testosterone

## Abstract

**Background:**

The hypothalamic–pituitary–adrenal (HPA) and -gonadal (HPG) axes are two major pathways that connect the neural and endocrine systems in vertebrates. Factors such as prenatal stress and maternal exposure to exogenous steroids have been shown to affect these pathways during fetal development. Another less studied factor is the transfer of hormones across fetuses in multifetal pregnancies. This form of transfer has been shown to influence the morphology, anatomy, physiology, and behavior of the offspring in litter-bearing mammals, an influence termed the intrauterine position (IUP) effect. In this study, we sought to delineate how the IUP effects HPA and HPG brain receptors, peptides, and enzymes (hereafter components) in utero and how these influences may differ between males and females.

**Methods:**

We utilized the unconventional model of culled free-ranging nutria (*Myocastor coypus*), with its large natural variation. We collected brain tissues from nutria fetuses and quantified the expression of key HPA and HPG components in three brain regions: prefrontal cortex, hypothalamus, and striatum.

**Results:**

We found an interaction between sex and IUP in the mineralocorticoid receptor (MR), gonadotropin-releasing hormone receptor (GNRHR), androgen receptor (AR), and estrogen receptor alpha (ESR1). IUP was significant in both gonadotropin-releasing hormone (GnRH) and its receptor GNRHR, but in different ways. In the hypothalamus, fetuses adjacent to same-sex neighbors had higher expression of GnRH than fetuses neighboring the opposite sex. Conversely, in the cortex, GNRHR exhibited the inverse pattern, and fetuses that were neighboring the opposite sex had higher expression levels than those neighboring the same sex. Regardless of IUP, in most components that showed significant sex differences, female fetuses had higher mRNA expression levels than male fetuses. We also found that HPA and HPG components were highly related in the early stages of gestation, and that there was an interaction between sex and developmental stage. In the early stages of pregnancy, female component expression levels were more correlated than males’, but in the last trimester of pregnancy, male components were more related to each other than female’s.

**Conclusions:**

This study suggests that there are sexually different mechanisms to regulate the HPA and HPG axes during fetal development. Higher mRNA expression levels of endocrine axes components may be a mechanism to help females cope with prolonged androgen exposure over a long gestational period. Additionally, these findings suggest different coordination requirements of male and female endocrine axes during stages of fetal development.

**Supplementary Information:**

The online version contains supplementary material available at 10.1186/s13293-024-00637-9.

## Highlights

- This study is the first to analyze HPA and HPG axes receptors, peptides, and enzyme mRNA expression levels in the brains of fetuses in the wild.
- Higher HPA and HPG axes receptors mRNA expression levels in females hint towards alternative sex-specific mechanisms that regulate endocrine axes during fetal development.
- Coordination of HPA and HPG axes components’ expression levels is different between males and females at different stages of gestation.

**Supplementary Information:**

The online version contains supplementary material available at 10.1186/s13293-024-00637-9.

## Plain language summary

In litter-bearing mammals, the positioning of a fetus in the uterus relative to other fetuses of the same or opposite sex has been shown to directly influence fetal morphology and physiology, and later behavior, reproductive success, and survival in adults. In this study, we sought to understand the mechanisms by which the location in the uterus influences two major neuroendocrine pathways in fetal brains. We quantified expression of multiple receptors and an enzyme (referred to as ‘components’) of the endocrine axes in three different brain regions in fetal free-ranging nutrias. Our results showed higher expression in females than in males for some endocrine axes components. The location inside the uterus was also related to the expression of some components. Lastly, coordination between the axes was higher earlier on in gestation, and females were more coordinated than males in the second trimester, whereas males were more coordinated than females in the third trimester. The results of this study point to the mechanisms by which the sexes regulate key neuroendocrine pathways during fetal development.

**Supplementary Information:**

The online version contains supplementary material available at 10.1186/s13293-024-00637-9.

## Introduction

The intrauterine environment has an extensive impact on the developing fetus, as well as pronounced long-term consequences on its reproductive success and survival. An important process that impacts fetal development involves steroids diffusing from fetuses to their neighbors in utero [7], reviewed by Fishman et al. [21], Ryan and Vandenbergh [63]. This phenomenon, termed the intrauterine position (IUP) effect, has been shown to influence morphological and physiological development, fetal maturation, sexual differentiation, and behavior in many litter-bearing (i.e., polytocous) mammals, including rats, mice, gerbils, nutrias (*Myocastor coypus*), and pigs [20, 21, 63, 76]. Most fetuses that develop next to males in utero exhibit masculinized traits [7], reviewed by Fishman et al. [21], Ryan and Vandenbergh [63]. For example, in mice, female fetuses that develop in utero between two males have up to 30% higher circulating testosterone concentrations than females located between two females [76]. The anogenital distance (AGD), i.e., the length of perineal tissue between the genitalia and anus, which is usually longer in males than in females, is also affected by IUP [21, 63, 76]. Female rodents that develop between two males in utero have a longer AGD than ones not adjacent to a male ([76], reviewed by Fishman et al. [21], Ryan and Vandenbergh [63]). In a dose–response study, prenatal administration of androgens to female rats induced male-like genitalia, elongated AGD, changes in the brain nuclei, and delayed puberty [79].

Steroid hormones are involved in the IUP effect because they are small, lipophilic molecules that are transferred not only through the circulation and amniotic fluid (AF) but also across membranes, such as those separating fetuses [7, 67, 76, 77]. Although most IUP research has examined the effects of testosterone transfer between fetuses, other steroid hormones also shape the physiology, morphology, and behavior of mammals. For example, glucocorticoids (GCs), such as corticosterone and cortisol, play a crucial role in preparing the fetus for extrauterine life [22, 52], including the promotion of lung maturation [42].

Steroid hormones are the products of the hypothalamic–pituitary–adrenal (HPA) and -gonadal (HPG) axes, two major signaling pathways connecting the neural and endocrine systems in vertebrates [1]. The two axes consist of several interacting components and mediators that contribute to the variation in the ability to produce steroid hormones [62]. Sex differences are found in many of the components, including peptides, receptors, enzymes, and binding proteins responsible for reproduction, dispersal, and other fitness-related behaviors [27, 61, 62]. Despite extensive research and knowledge on IUP and sex differences along the HPA and HPG axes, little is known about how the IUP effects the components of the axes, and how these effects differ between males and females.

To test whether the sex of neighboring fetuses affects HPA and HPG receptors, peptides, and enzymes (hereafter components), we measured the effects of IUP and sex on mRNA expression levels in the brains of free-ranging culled nutria fetuses. The nutria is an excellent model because of its long gestations (up to 140 days), large litters (up to 13 fetuses, average of 6), precocious offspring with large, developed brains, and year-round breeding. Fetal sexing is possible by PCR (for fetuses < 75 days gestation, using the SRY gene) or by observing internal and external anatomy (See Fig. 1S in Supplementary Information).

Male nutria fetuses have higher testosterone levels than female fetuses, but contrary to lab rodents, female fetuses next to males have the lowest testosterone levels in the litter, and female fetuses that do not neighbor a male have similar testosterone levels to males [21]. No sex differences were found in fetal cortisol levels, but nutria fetuses neighboring an opposite-sex fetus in utero have higher cortisol levels and longer trunks [20], indicating an advantage of high cortisol levels in utero. Moreover, litters with a higher proportion of fetuses neighboring an opposite-sex fetus have higher levels of cortisol, after correcting for litter size [20]. As steroids operate through receptors and endocrine axes moderators, understanding how sex and IUP associate with HPA and HPG axes components in utero can reveal the evolutionary forces shaping sex differences and their implications.

## Results

Since nutrias are invasive species that are culled by the authorities, we followed hunters and collected female nutria carcasses in the field. Approximately 80% of adult females were pregnant (see Methods).

### Identification of nutria HPA and HPG axes gene sequences

**Fig. 1:**
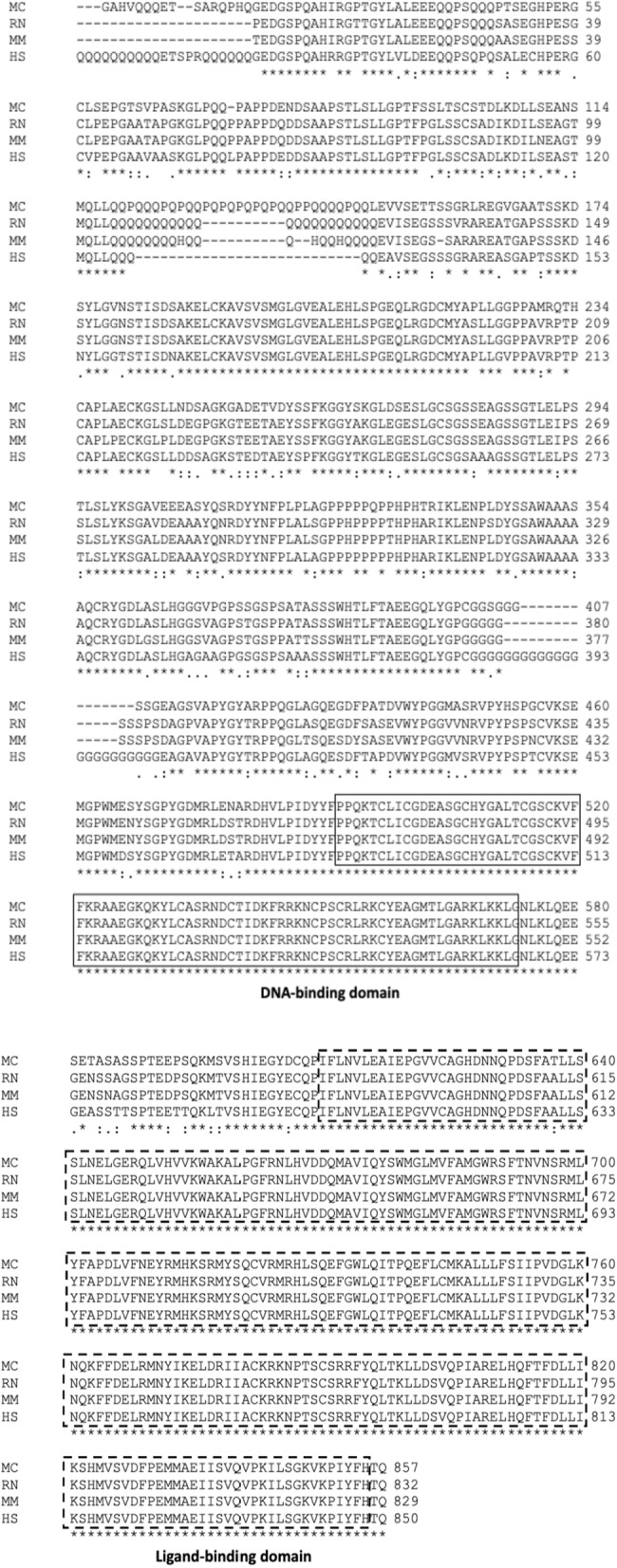
Multiple sequence alignment (MSA) of the AR protein sequence identified in nutria, with rat (*Rattus norvegicus*; RN; NP_036634.1), mouse (*Mus musculus*; MM; NP_038504.1) and human (*Homo sapiens*; HS; NP_000035.2) annotated sequences. Identical amino acids are marked with an asterisk (*), highly conserved amino acids are marked with a colon (:), and conserved amino acids are marked with a period (.). The DNA-binding domain is marked within continuous lines, and the ligand-binding domain is marked within dashed lines

We identified the sequences for the HPA (GR and MR) and HPG (GnRH, GNRHR, AR, aromatase, and ESR1) axes genes in nutria by way of bioinformatic analysis. Identified genomic coordinates and their corresponding coding sequences for HPA and HPG axes components are documented in Supplementary Information (SI) Tables 1A and 1B, and the mRNA and protein sequence IDs of their rat ortholog are listed in SI Table 1C. The HPA and HPG axis components’ sequences were highly conserved across species (Table 1; the protein sequences used for the analysis of homology are in SI Table 2). For example, the sequence alignment of AR in nutria is highly homologous to AR sequences in other species, including the DNA and ligand-binding domains (Fig. 1).

**Table 1:** Percent similarity and coverage (in parentheses) of *Myocastor coypus* (nutria) sequences to *Rattus norvegicus* (rat), *Mus musculus* (mouse), and *Homo sapiens* (human) sequences

Organism	Protein	*Rattus norvegicus*	*Mus musculus*	*Homo sapiens*
*Myocastor coypus*	GnRH	82 (95)	85 (92)	84 (100)
	GNRHR	90 (100)	91 (100)	92 (100)
	AR	89 (100)	88 (100)	91 (77)
	aromatase	89 (100)	90 (100)	89 (100)
	ESR1	91 (100)	92 (100)	91 (100)
	GR	91 (100)	91 (100)	93 (100)
	MR	94 (100)	95 (100)	95 (100)

*GnRH* gonadotropin releasing hormone, *GNRHR* gonadotropin releasing hormone receptor, *AR* androgen receptor, *ESR1* estrogen receptor alpha, *GR* glucocorticoid receptor, *MR* mineralocorticoid receptor

### Significant sex and IUP differences in HPG axis components’ expression levels

**Fig. 2:**
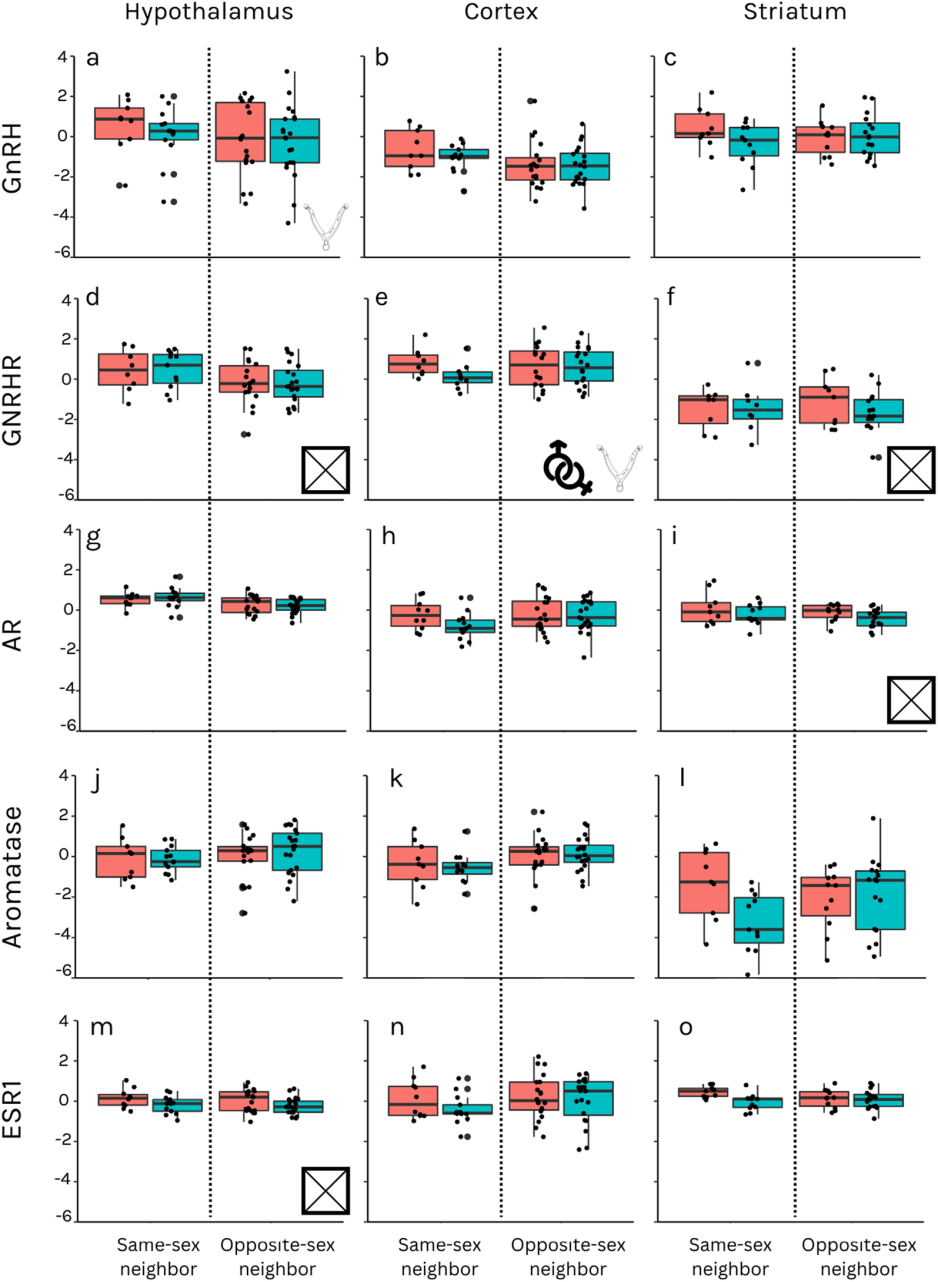
HPG axis components’ mRNA relative expression levels in the hypothalamus (**a**, **d**, **g**, **j**, and **m**), prefrontal cortex (**b**, **e**, **h**, **k**, and **n**), and striatum (**c**, **f**, **i**, **l**, and **o**) of nutria fetuses. Each row represents the relative expression of a gene, and each column a brain region. Blue box plots represent males and red box plots females. Each point on the graph represents an individual fetus. The y-axis of each graph is the relative expression of each component. Significant results are represented using icons (sex symbols = significant sex result; uterus symbol = significant IUP result; X symbol = significant sex and IUP interaction) located on the bottom right of the relevant gene and brain region. In graphs a and e, GnRH and GNRHR expression is higher in fetuses neighboring the opposite sex than those neighboring only same sex fetuses. In graphs d, f, i and m, there is a significant interaction between sex and IUP. In graph e, females have higher GNRHR expression than males

We found that female fetuses in the last trimester had higher GNRHR mRNA expression levels in the prefrontal cortex than male fetuses (β = 0.186; F = 5.409; p = 0.036; Fig. 2e; SI Table 3; with hair testosterone as a model effect). GNRHR expression levels in the prefrontal cortex were higher in fetuses neighboring the opposite sex than those neighboring a fetus of the same sex (β = − 0.231; F = 5.943; p = 0.032; Fig. 2e, SI Table 5; with AF testosterone as a model effect). IUP was also associated with GnRH expression in the hypothalamus (β = − 0.627; F = 17.553; p = 0.003; Fig. 2a, SI Table 4), so that fetuses with same sex neighbors had higher mRNA expression levels than those that neighbored a fetus of the opposite sex.

An interaction between sex and IUP was found in hypothalamic GNRHR mRNA expression levels (β = − 0.433; F = 9.034; p = 0.011; Fig. 2d, SI Table 4). Females neighboring males had higher GNRHR expression than those neighboring only females, but males adjacent to females had lower GNRHR expression than males that only neighbored males. This is consistent with results from the striatum (β = − 0.638; F = 7.941; p = 0.023; Fig. 2f, SI Table 6).

In the third trimester, the interaction between sex and IUP explained AR expression in the striatum (β = 0.231; F = 4.907; p = 0.043; Fig. 2i, SI Table 7), so that only in fetuses that had neighbors of the opposite sex, females had higher mRNA expression than males. In addition, sex and IUP interacted to explain ESR1 mRNA expression levels in the hypothalamus (β = − 0.209; F = 13.611; p = 0.002; Fig. 2m, SI Table 8).

### Significant sex and IUP differences in the HPA axis components’ expression levels

**Fig. 3:**
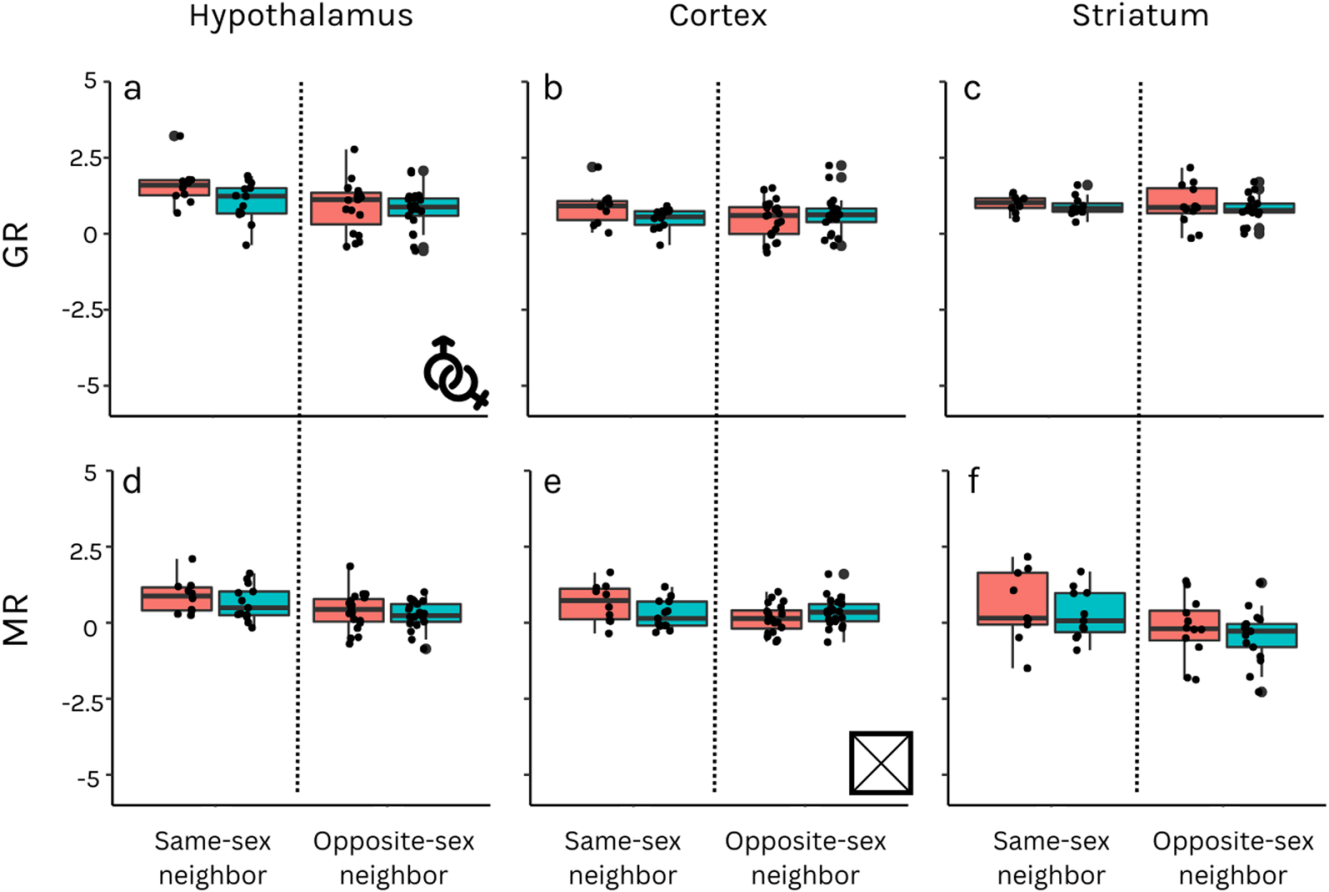
HPA axis components mRNA relative expression levels in the hypothalamus (**a**, **d**), prefrontal cortex (**b**, **e**), and striatum (**c**, **f**) of nutria fetuses. Each row represents the relative expression of a gene, and each column a brain region. Blue box plots represent males and red box plots females. Each point on the graph represents an individual fetus. The y-axis of each graph is the relative expression of each component. In graph a, females have higher GR expression than males. In graph e, there was a significant interaction between sex and IUP. Significant results are represented using icons (sex symbols = significant sex result; X symbol = significant sex and IUP interaction result) located on the bottom right of the relevant gene and brain region

We found that females had higher GR mRNA expression levels in the hypothalamus than males (β = 0.2185; F = 6.982; p = 0.014; Fig. 3a, SI Table 9). In addition, the interaction between sex and in utero location was significant in predicting MR mRNA expression levels in the prefrontal cortex (β = 0.164; F = 7.611; p = 0.014; Fig. 3e, SI Table 10) in fetuses in the last trimester of gestation (> 110 days). Females that only neighbored females in utero had higher MR expression levels than female fetuses with a male neighbor. In males, the opposite was found, and males with only male neighbors had lower MR expression levels than male fetuses that had a female neighbor. We did not find significant effects in other brain regions (SI Tables 11–14).

### Sex differences in the coordination between HPA axis and HPG axis components during gestation

**Fig. 4:**
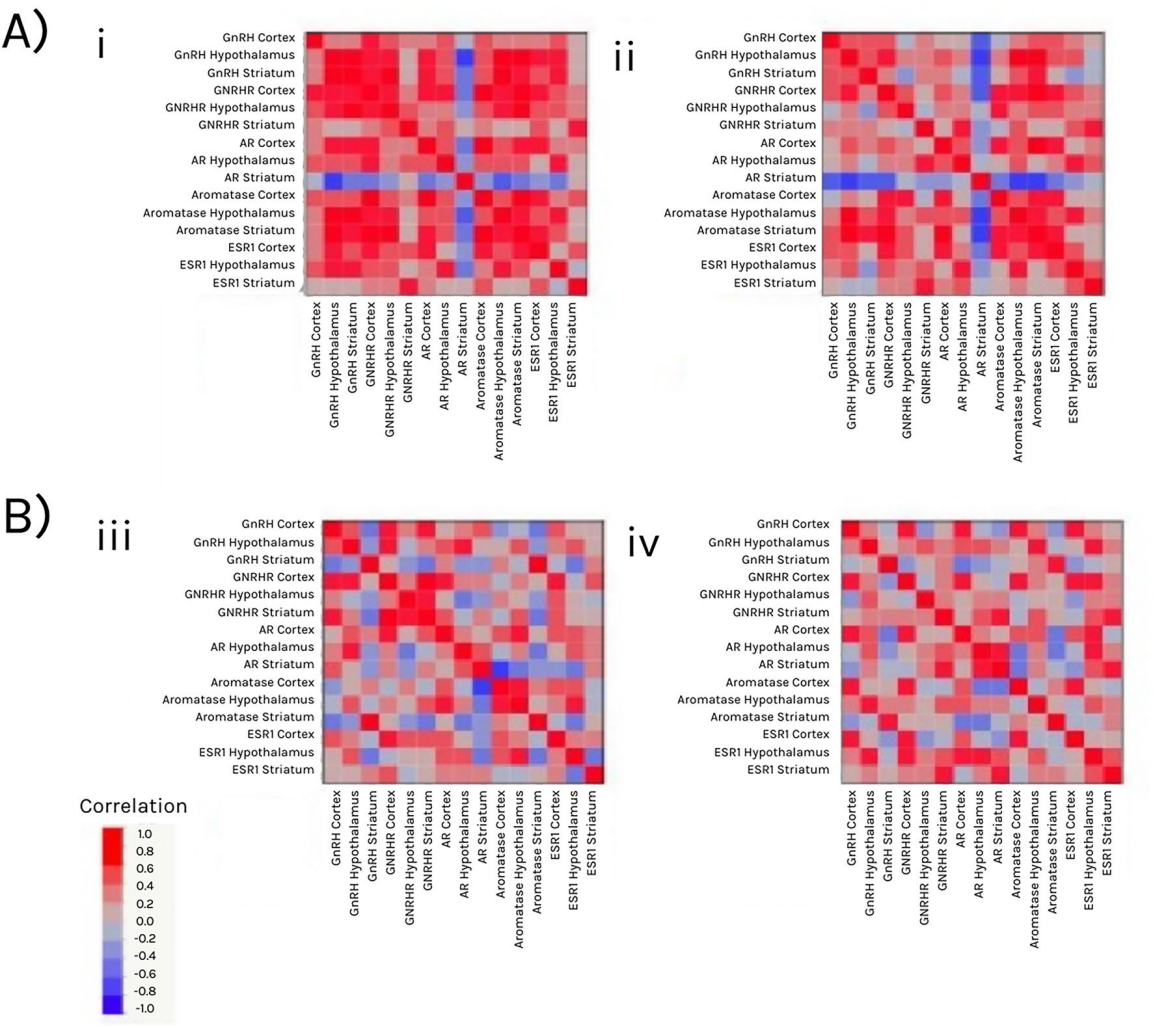
Comparison of correlation patterns between HPA and HPG components in three brain regions during the second trimester (**A**) and the third trimester (**B**). Saturated colors denote a higher positive correlation efficient (red) or a higher negative correlation efficient (blue). The androgen receptor (AR) in the striatum is negatively correlated with many genes earlier in development, i.e., during the second trimester. Genes that did not correlate are in a light (pink or blue) color

**Fig. 5:**
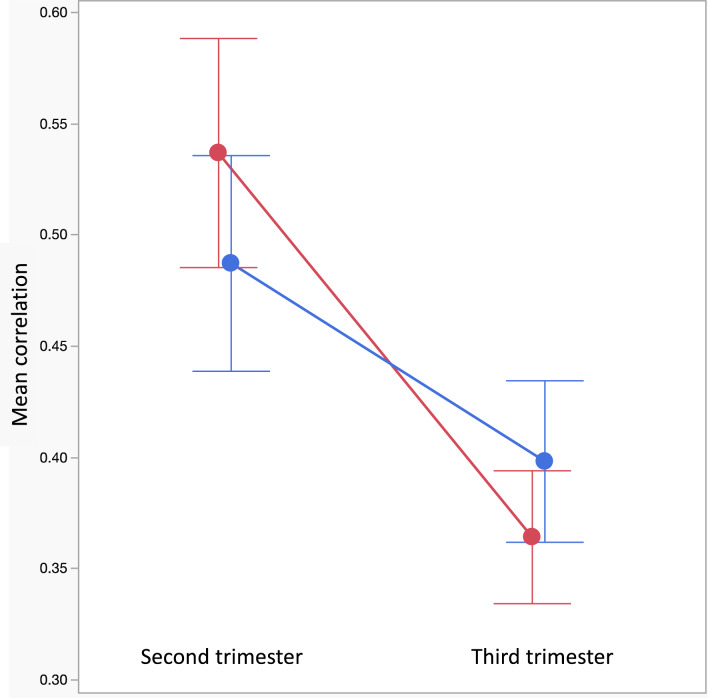
Comparison of correlations between HPA and HPG axis components in male (blue) and female (red) fetuses. Overall, components are more correlated in the second trimester than in the third trimester. In the second trimester, female components are more correlated than male components, while in the third trimester male components are more correlated than female ones

We compared the relationship between gene expression profiles across brain regions of male vs. female fetuses in the second and third trimesters of gestation (Fig. 4). Our results indicate that HPA and HPG axis components in both sexes are more coordinated in the second than in the third trimester (F_1_ = 41.343; P < 0.0001). Although there are no significant sex differences in the correlations between the components, we found a significant interaction between sex and developmental stage (F_1_ = 4.222; P = 0.0432; Fig. 5). Females’ components are more highly correlated than males’ components earlier on in gestation (i.e., in the second trimester), while the male components are more highly correlated than the females later in gestation (i.e., in the third trimester).

## Discussion

We found that mRNA expression levels of most HPG and HPA axes receptors and proteins that were quantified were higher in female fetuses relative to males. Higher brain receptor expression levels may serve as a regulatory mechanism, especially when circulating hormone concentrations are low. For example, in adult African black coucals (*Centropus grillii*) and rock doves (*Columba livia*), although males had significantly higher circulating androgen concentrations than females, females had higher AR mRNA expression levels than males [44, 75]. In the present study, mRNA expression levels were quantified in fetal brains, suggesting upstream organizational effects shaping the morphology, physiology and behavior of adults. Further, the sex of neighboring fetuses in utero interacted with fetal sex to influence HPG and HPA axes expression levels. In some cases, HPG axes receptors and protein mRNA expression levels were higher in fetuses neighboring a fetus of the opposite sex (P1) compared to those neighboring a same-sex fetus (P0), and in others, the opposite was seen. For example, females neighboring only female fetuses (P0) had higher AR mRNA expression levels but lower ESR1 mRNA expression levels than those neighboring male fetuses (P1). There were less differences between males with or without female neighbors. These results support our previous findings that females neighboring only females in utero (P0) had the same hair testosterone levels as those of males, which were higher than testosterone levels of female fetuses that neighbored a male in utero (P1; [21]). The opposite trends between AR and ESR1 may reflect the aromatization of androgens to estrogens during late gestation. Lower circulating testosterone in females neighboring males in utero may be compensated by higher AR and may be a mechanism that evolved in the nutria to avoid the detrimental costs associated with chronic exposure to high circulating testosterone concentrations over the course of a long gestation (~ 140 days). IUP studies assume testosterone transfer between fetuses (i.e., neighboring a male in utero increases testosterone exposure). Indeed, our analysis of fetal nutria AGD showed that both sexes were exposed to increased testosterone during early development when neighboring a male fetus [21]. However, our results indicate that for both males and females, neighboring a fetus of the opposite sex is associated with an increase in HPG axis mRNA expression levels. Thus, it is not likely testosterone concentrations but the presence of the opposite sex that turns on HPG-axis-related genes.

Proximity to a fetus of the opposite sex was also related to mRNA expression levels of receptors along the HPA axis. We found that GR mRNA expression levels in the hypothalamus were higher in females than in males. The IUP interacted with sex in opposite ways to influence MR mRNA expression levels in the cortex. While females that only neighbored females in utero had higher MR mRNA expression levels than those neighboring a male, males that neighbored a female had higher mRNA expression levels than those that were only next to males. Although the IUP has been linked to fitness, with implications that carry into adulthood [63], the transfer of cues and glucocorticoids between fetuses themselves, via the IUP effect, has not been investigated. Glucocorticoids may bind to the MR in utero to affect systems that regulate fetal growth, including the development of cardiovascular, metabolic, reproductive, and neurological function [52]. Therefore, the lower MR expression levels in female nutria fetuses neighboring a male in utero may indicate a lower sensitivity and a possible reduction in HPA axis activity to counteract increased glucocorticoid levels over the course of gestation. This may serve as a fetal protective mechanism to counteract the potentially detrimental effects of glucocorticoid overexposure, which may disrupt the development of crucial and sensitive functions.

Our results suggest that glucocorticoid exposure over the course of gestation modulates fetal MR, and not the GR, and that baseline levels are regulated via the MR. Most research on sex differences along the HPA axis measure cortisol and GR levels as a function of maternal and/or fetal stress. For example, female rodents typically have a more elevated response to acute stress compared to males (reviewed by Heck and Handa [26]). In humans, although baseline cortisol concentrations are typically comparable between men and women, there are sex differences in cortisol following stress (reviewed by Bangasser and Valentino [6]). Previous studies on sex differences along the HPA axis suggest that environmental factors such as maternal stress, nutrition, and bioactive substances play an important role in shaping axis reactivity. Over the course of gestation, glucocorticoids aid in transferring maternal cues to the fetus (reviewed by Marciniak and Patro-Ma [46]), with maternal stress (reviewed by Weinstock [78]), for example, altering female, but not male, body weight and reproductive parameters [65]. In the present study we did not have information about fetal or maternal stress. However, we found sex differences in fetal HPA axis activity that may be facilitated by gonadal hormones during key developmental periods (e.g., [66], reviewed by Handa and Weiser [25], and Heck and Handa [26]).

Lastly, in the present study, we found that female HPA and HPG axes components during the second trimester were more synchronized than those of males, while in the third trimester, male axes components were more correlated than those of females. Although it remains unclear what causes the shift in correlation from early to late gestation, the higher correlation between gene expression levels earlier in gestation may indicate a critical period in the development of both the HPA and HPG axes during or before the second trimester. Critical periods are documented across species, yet not all brain regions follow the same developmental time course, and different brain circuits develop when their function is required for receiving and processing signals [28]. Likewise, steroid hormone action on the brain during fetal development occurs in specific, hormone-sensitive developmental periods [4], and the degree of sex-biased gene expression has been shown to generally amplify over the course of development [14], reflecting an increase in phenotypic dimorphism. Axis coordination may be needed for biological processes related to development and homeostasis. Regulation of HPA and HPG axis components’ expression during fetal development is important to ensure the proper coordination of tissue growth and differentiation and the orderly maturation of vital organ systems. This process is crucial for synchronizing the appropriate timing of parturition to successfully transition a fetus from intrauterine to extrauterine life [56]. Towards the end of gestation, there may be a shift towards growth, to prepare the fetus for extrauterine life, possibly explaining the reduction in coordination between HPA axis and HPG axis components in the different brain regions.

At each tier of the HPA and HPG axes, along the potential for functional variation of the amount of hormone secreted, there is also functional variation in the sensitivity to hormones secreted by other tiers and variables. Little is known about how well sex-biased gene expression corresponds to sex-biased protein synthesis [16], since transcript abundance is not synonymous with receptor levels. For example, post-translational processes can compensate for increased synthesis by increasing the rate of protein turnover [68]. Moreover, gene expression bias can vary between studies of the same species, which can be due to tissue specificity, developmental stage, genetic and environmental variation, and the experimental design [30]. Gene expression bias may be further amplified in wildlife, due to natural genetic and environmental variation between individuals of the same species.

Although steroid hormones influence development in all vertebrates [1] and the function of the HPA and HPG axes is generally conserved [44], we found that the sex, IUP, and their interaction varies. Steroid hormone receptor mRNA expression levels may be regulated independently of gonadal hormones and glucocorticoids and/or modulated by steroid synthesis in the brain. Variability in the cellular and molecular properties of target tissues that include measures of sensitivity to steroid hormones are critical to explaining the functional variation in behavior, physiology, and development [61]. However, whether sex-biased gene expression is a cause or a consequence of sexual dimorphism is unknown. Further studies in wildlife may help elucidate the complex mechanisms by which the HPA and HPG axes are modulated during fetal development.

### Perspectives and significance

This study is the first to measure wildlife endocrine axes expression levels in fetal brains. The results suggest sex- in utero location- and developmental stage-specific regulation of key features, with overall higher female gene expression levels. The wild nutria is an excellent model to study sex differences and IUP effects in fetuses due to its long gestation, resembling human pregnancies more than traditional laboratory rodent models such as mice and rats. The systematic patterns found in these free-ranging mammals that exhibit high genetic and environmental variation suggest that they are robust. Future directions include RNAseq analysis that will include additional endocrine components and assessments of sex differences in component heritability.

## Methods

### Sample collection and sample size

**Fig. 6:**
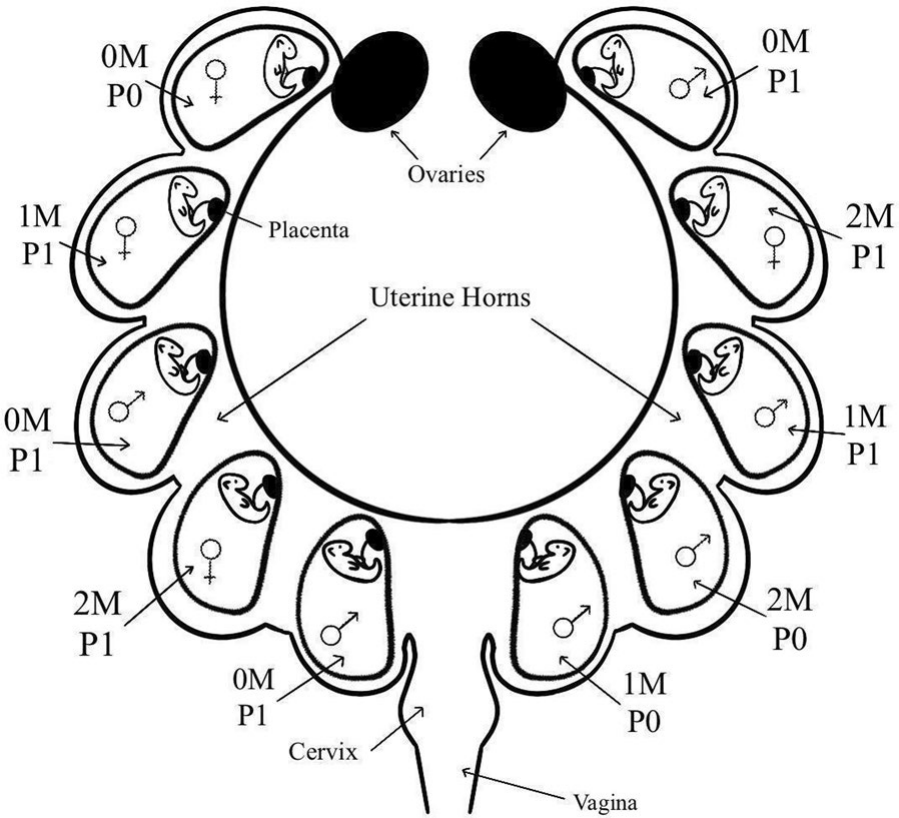
IUP labeling example using two conventions. The Contiguity Method (vom Saal et al. [76]) refers to a fetus’ position relative to males. 0 M = fetus not next to a male; 1 M = fetus neighboring one male; 2 M = fetus neighboring two males. The second convention refers to a fetus’ position relative to a fetus of the opposite sex [19]. P0 = fetus next to fetuses of their own sex, P1 = fetus neighboring a fetus of the opposite sex on either or both sides. Figure designed by Ariel Yael, drawn by Aiden Braner

Nutrias were collected through the culling efforts of local authorities (Jewish National Fund–JNF–KKL) by professional hunters using shotguns at the Hula Lake Park, Israel over several campaigns from December 2019 to March 2021. We followed the hunters, collected the culled nutrias, and dissected them within 30 min. Since nutria are an invasive species in Israel and are not protected by law, permits were not required for collecting carcasses. In this study, a total of 27 pregnant females (with a gestation age of 60–131 days) and their fetuses (N = 153) were included. Carcasses of pregnant females were weighed using a spring scale (10 kg, Pesola, Switzerland) and morphometric measurements (e.g., total length, nose to the base of the tail, shoulders to the base of the tail, tail length, tail base circumference, and right hind foot) were conducted using a measuring tape to the nearest millimeter. Upon dissection, the IUP for each fetus was determined, including the uterus horn location (left or right), and its location relative to the ovary (the closest fetus to the ovary was numbered one). Moreover, two conventions were used to label fetuses according to their proximity to other fetuses (Fig. 6). Fetuses were sexed according to external morphology using AGD (see Fig. 1, [19]). Fetuses in litters with an estimated gestational age of > 60 days were weighed using an analytical balance to the nearest 0.01 mg (Precisa, Switzerland). The gestational age was estimated using Newson’s formula [55]: Estimated gestational age = $43.69+14.27\times \sqrt[3]{average\, fetal\, weight}$

### Tissue isolation and storage

**Fig. 7:**
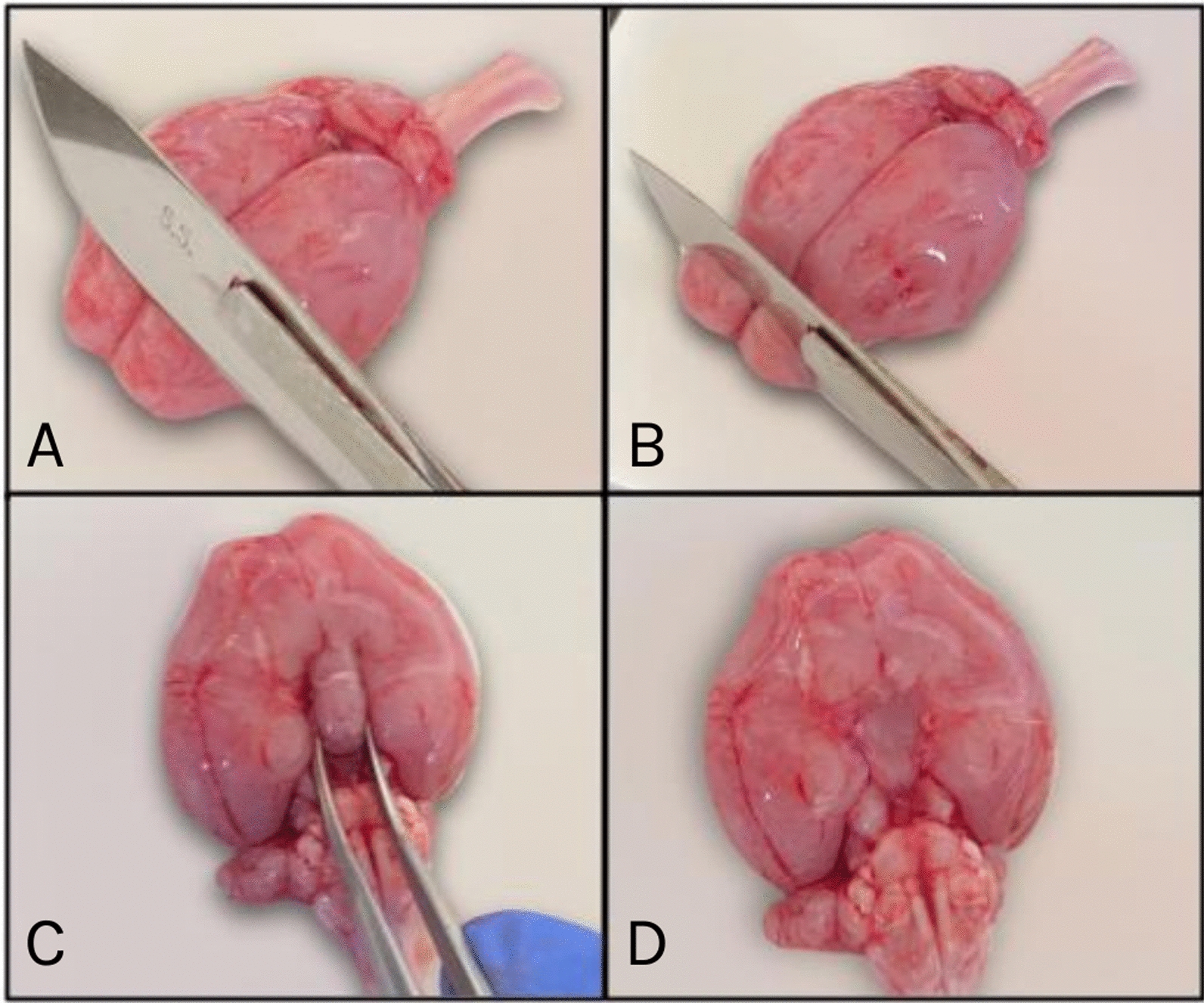
Fetal nutria brain dissections. **A** Nutria whole fetal brain, ventral view; **B** Prefrontal cortex was dissected with a scalpel, at an approximately 45º angle; **C** Hypothalamus, located on the ventral side of the brain, was removed with sharp tweezers (**D** once removed); Not pictured: the striatum

Whole brains were isolated from 64 fetuses (11 litters, with a gestational age of > 85 days) and grossly dissected into the hypothalamus, prefrontal cortex, and striatum (Fig. 7). Samples were placed in sterile cryogenic tubes, snap-frozen with liquid nitrogen for several hours, and stored at − 80 ºC until analysis.

### RNA extraction and RNA quality analysis

Frozen brain samples (tissue weight: 22.56–49.99 mg) were thawed on ice and homogenized for 30 s using TissueLyser II (Qiagen, Germany) with two sterile 2.5 mm stainless-steel beads in 500 µL of TRIzol reagent (Bio-Lab Ltd., Israel). Following homogenization, total RNA was extracted with TRIzol according to the manufacturer’s protocol. RNA yield and purity, analyzed by the OD A260/A280 and A260/A230 ratio, were determined by the NanoDrop 2000 Spectrophotometer (Thermo Scientific).

### Sequence analysis for primer design

Like the analysis detailed by Matas et al. [47], assembled nutria genome sequences were downloaded from the NCBI site (https://www.ncbi.nlm.nih.gov/assembly/GCA_004027025.1/) and used to create a BLAST database. Using tBLASTx, *Rattus norvegicus* HPA (GR and MR) and HPG (GnRH, GNRHR, AR, aromatase, and ESR1) axes genes were used as a query to identify sequences of corresponding nutria orthologs (SI Table 1C). The best hit both in terms of coverage and similarity was selected. Seqret from the EMBOSS package was used to extract the corresponding genomic loci. The mRNA sequence was predicted using GENSCAN, then Splign was run using the predicted mRNA sequence and the corresponding genomic loci to ascertain the exact exon–intron splice sites. Identified coding sequences for HPA and HPG axes components are documented in SI Table 1B. The housekeeping gene glycogen synthase 1 (Gys1) was also identified (SI Table 15). Identified protein sequences of these nutria genes were analyzed by multiple sequence alignment (MSA) with other homologs using CLUSTAL Omega algorithm. Similarity and coverage were determined by NCBI blast. Identification of known domains was carried out by NCBI Search for conserved domains within a protein.

### Reverse transcription and real-time PCR

Genomic DNA was removed from nutria fetal brain RNA samples by DNA-*free* DNA Removal kit (Invitrogen, Thermo Fisher Scientific, MA, USA), and reverse transcription of the purified RNA was performed using the qScript cDNA Synthesis Kit (Quantabio, MA, USA). The resulting cDNA was used as a template for real-time PCR (qPCR) with CFX- 96 (Bio-Rad Laboratories, Hercules, CA, USA) using SYBR Green FastMix/ROX qPCR Master Mix (Quantabio, MA, USA), according to the manufacturer’s instructions. We measured mRNA expression levels of the genes for GR, MR, GnRH, GNRHR, AR, aromatase, and ESR1 in the prefrontal cortex, hypothalamus, and striatum. We also quantified the expression of the housekeeping gene Gys1 for normalization of target genes. Forward and reverse primer sequences are reported in SI Table 16. Primers were synthesized by Syntezza Bioscience Ltd. (Jerusalem, Israel) from exon–intron junction sequences. All primers were validated for non-specific amplification and primer-dimer formation by melt curve analysis. Efficiencies of all primers range from 90.3 to 100.5%. qPCR reactions were run in triplicates using cDNA diluted according to the validations. Thermocycling conditions were: 95 ℃ for 30 s and 55 ℃ for 30 s, for 40 cycles. A final melting phase of 65–95 ℃ (by increments of 0.5 ℃) for 30 s was run to confirm the single-product specificity of each sample.

### Hair testosterone and cortisol extraction and quantification

Using hair to quantify steroids provides a long-term steroid accumulation profile over the time of growth and reflects free, unbound, and presumably bioavailable hormones [37]. Moreover, hair steroid levels are not sensitive to the acute stress of culling [19, 37]. This method has been validated in this system as an indicator of maternal status during pregnancy and of the condition of the fetuses in utero [19–21]. Fetal hair follicles in the nutria appear at 85–90 days of gestation, around the beginning of the last trimester of their ~ 130 day-long gestation [21]. Hair samples were collected from 25 fetuses past the gestational age of 111 days. As detailed in Fishman et al. [21], all fetuses were washed under the tap with deionized water to remove blood and amniotic fluid, then dried for at least 24 h. Once dried, hair was shaved from the entire fetus using an electric razor (Moser Chromini 1591 0.7 mm). Hair samples were washed twice with isopropanol (Romical, Israel) on a shaker for three minutes to remove external contaminants and dried again for a minimum of 24 h in an open petri dish (10 mm). Dried hair samples were weighed, and steroids were extracted by methanol (Romical, Israel), sonicated for 30 min in a sonication bath (Elma, Germany), and then incubated overnight at 50 ℃ for 19 h with gentle shaking. Methanol was then separated from hair, centrifuged to remove small particles, and dried under a stream of nitrogen gas in a Dry-block heater at 45 ℃ (Techne, UK).

Hair cortisol and testosterone were quantified with a commercial enzyme-linked immunosorbent assay (ELISA) kit (Salimetrics, Europe, Newmarket, UK) using a protocol that was developed for wildlife and previously validated for nutria [19–21]. For testosterone, the manufacturer reported antibody cross-reactivity of 36.4% with dihydrotestosterone, 21.02% with 19-nortestosterone, 1.9% with 11-hydroxytestosterone, 1.157% with androstenedione, and < 0.49% with all other steroids. For cortisol, reported antibody cross-reactivity was 19.2% with dexamethasone and < 0.568% with all other steroids. Validations showed linearity (in the range of 5–40 mg hair for testosterone and 0.5–10 mg hair for cortisol) and parallelism between serially diluted hair extracts and kit standards (slope covariance P = 0.91 for testosterone and P = 0.36 for cortisol). Intra-assay CV for six repeats on the same plate was 7.23% for testosterone and 5.29% for cortisol. Inter-assay CV was 0.6% for testosterone across four plates and 13.1% for cortisol across five plates. Recovery was quantified by spiking hair samples with a known steroid amount and was calculated as 100.7% for testosterone and 90.9% for cortisol.

### LC–MS/MS validation

The presence of cortisol and testosterone in a pooled nutria hair extract was confirmed via LC–MS/MS using a similar sample preparation method as a recent study of cortisol in muskox qiviut [15]. The hair pool was gently washed by hand rotation in 20 mL ice-cold tap water with Neutrogena TM water for 3 min and then rinsed by tap water for 0.5 min. After being quickly rinsed by 20 mL ice-cooled HPLC grade IPA, the sample was dried by paper towel and placed in a fume hood overnight. The sample was then transferred into a 13 × 100 mm culture test tube. As an additional validation, another test tube contained 200 mg of the burial matrix that surrounded the individual hair samples. To both test tubes, 100 μL of deuterium labeled internal standard solution and 9 mL cold methanol were added. The test tubes were capped and stored in a 4 ℃ fridge for 20 h. After the sample was removed from the test tube, the extract was evaporated to dryness under N2 at 40 ℃ by use of Techne Sample Concentrator and reconstituted with 150 µL of H2O/MeOH (50/50, v/v). The solution was centrifuged at 14,000 rpm (Legend micro-21R, Thermo Scientific) for 20 min and 120 µL of supernatant was submitted to LC–MS.

All samples were analyzed by using an Agilent 1200 binary liquid chromatography (LC) system connected with an AB SCIEX QTRAP^®^ 5500 tandem mass spectrometer equipped with an atmospheric pressure chemical ionization (APCI) source. LC separation was performed on an Agilent Poroshell 120 C18 column (50 × 3 mm, 2.7 µm particle size) at 45 ℃. The mobile phase A was H2O/MeOH (75/25, v/v) and the mobile phase B was MeOH/IPA (90/10, v/v). The 8.5 min gradient was 20–40% B (0–1.0 min), 40–60% B (1.0–5.0 min), 60–100% B (5.0–5.5 min), 100% B (5.5–6.5 min), 100–20% B (6.5–7.0 min), and held at 20% B (7.0–8.5 min). The flow rate was 0.6 mL/min and the injection volume was 20 µL. The analytes were ionized under positive APCI mode and data were acquired via multiple reaction monitoring (MRM). The lowest limit of quantification for cortisol was 0.25 ng/mL and for testosterone 0.05 ng/mL, which were the lowest concentrations that gave < 20% CV and <  ± 20% error. Nutria hair pool steroid levels showed 20.26 pg cortisol/mg hair and 0.51 pg testosterone/mg hair. More details about the quantification protocol are provided in the SI (Tables 17–19).

### Amniotic fluid (AF) testosterone and cortisol extraction and quantification

AF was sampled from 52 fetuses (8 litters, with gestational age > 85 days) whose amniotic sac was intact. AF was extracted from the amniotic sacs of each individual fetus using a sterile syringe and needle. AF samples were flash frozen in liquid nitrogen and stored in -80ºC until analysis. Cortisol was quantified in AF samples following dilution in assay diluent, which was provided with the commercial ELISA kit (see below). For testosterone measurement, AF samples were extracted with ethyl acetate, vortexed, and centrifuged at 13,300 g for 10 min. The supernatant was collected and evaporated under a stream of nitrogen. Samples were then reconstituted in 10% methanol and 90% assay diluent.

Testosterone and cortisol were quantitated in AF extracts using commercial ELISA, according to the manufacturer’s recommendations (Salimetrics; Ann Arbor, MI, USA, item no. 1-3002-5, for cortisol, and item no. 1-2402-5 for testosterone). The cross-reactivity of the kits is reported above, in the quantitation in hair section. Testosterone validation using serial dilutions of AF pool (N = 20) showed linearity between 10 and 200 µL AF (equivalent to 15–240 pg/ml testosterone) and parallelism with the provided kit standards (univariate analysis of variance in SPSS; P = 0.805). Intra-assay repeatability was determined using 6 replicates of the pool on the same ELISA plate. The calculated coefficient of variation was 12.6%. Inter assay variability was determined by running duplicates of the pool on 4 different plates (n = 8). The coefficient of variation was 18.32%. Efficiency of 116.44% was retrieved using exogenous testosterone. Serial dilutions of AF pool (n = 12 samples) for cortisol validation showed linearity between 2 and 22 µL AF (equivalent to 0.111–3 μg/dL cortisol). Dilutions of the pool were parallel to the kit standards (univariate analysis of variance in SPSS; P = 0.071). Intra-assay repeatability using 6 replicates of the pool on the same ELISA plate showed coefficient of variation of 6.546%. Inter assay variability was determined by running 6 duplicates of the pool (n = 12). The coefficient of variation was 7.11%. Recovery of 84.5% was demonstrated using exogenous cortisol.

### Statistical analyses

All statistical analyses were performed using the JMP software (v.16). We used general linear mixed models (GLMM) with standard least squares to test whether sex (M/F), IUP (P0/P1), and their interaction (sex*IUP) affect HPA and HPG axes components separately for each brain region. We tested normality with Shapiro-Wilks tests, and when necessary, we used a log transformation (hypothalamus: GnRH, AR, ESR1, GR, and MR; cortex: GnRH, GNRHR, AR, aromatase, GR, and MR; striatum: GNRHR, AR, aromatase, ESR1, and MR). When the log-transformed values were still not normally distributed, the SHASH transformation (hypothalamus: GNRHR; cortex: ESR1; striatum: GnRH, MR) or the Johnson Sb transformation (hypothalamus: aromatase) were used.

Maternal identity and year were included as random factors in all models to account for variability between individual mothers and sampling over the 2 years (2020 and 2021). Litter size and season were included in all models. Estimated gestational age and fetal weight are interchangeable variables since the calculation of estimated gestational age depends on the average fetal weight for each litter [55]. For HPG axis components, we tested models including either estimated gestational age, fetal weight, or a residual of estimated gestational age and fetal weight, along with litter size and season, using model selection (AICc value; Tables SI 20–22). For HPA axis genes, we tested models including either estimated gestational age, fetal weight, a residual of estimated gestational age and fetal weight, or shoulders to the base of the tail length (SBL), or a residual of SBL and estimated gestational age, along with litter size and season, using model selection (AICc value; Tables SI 23–25). Fishman et al. [20] observed that nutria fetuses neighboring an opposite-sex fetus in utero were longer from shoulder to base of the tail, regardless of sex, implicating better lung development. Therefore, for analyses of HPA axis components (i.e., GR and MR mRNA expression level), we included SBL in the model criteria test.

In a subset of fetuses in the last trimester of gestation (i.e., with hair samples), we tested whether HPA axis components expression is affected by sex, IUP, sex*IUP, and fetal hair cortisol levels and whether HPG axis components expression is affected by sex, IUP, sex*IUP, and fetal hair T levels. To test the bidirectional interaction between the HPA and HPG axes, we analyzed the effect of sex, IUP, sex*IUP, and fetal hair T levels on HPA axis components and sex, IUP, sex*IUP, and fetal hair cortisol levels on HPG axis components.

To examine the relationship between gene expression across brain regions, we analyzed pairwise correlations using Pearson’s correlation coefficient (R). Correlation tables for all genes in all brain regions were split by age according to fetal weight and SBL averages (Group 1: 85–110 days; Group 2: 111–130 days). The split was verified using Tukey–Kramer analysis that showed significant differences between developmental groups.

### Supplementary Information

Supplementary Material 1.

## Data Availability

All data used in the study appears in the Supplementary Materials.

## References

[1] Adkins-Regan E. Hormones and animal social behavior. Princeton University Press; 2005.

[2] Arnold AP. A general theory of sexual differentiation. J Neurosci Res. 2017;95(1–2):291–300. 10.1002/jnr.23884. 27870435 10.1002/jnr.23884 PMC5369239

[3] Báez-Mendoza R, Schultz W. The role of the striatum in social behavior. Front Neurosci. 2013. 10.3389/fnins.2013.00233. 24339801 10.3389/fnins.2013.00233 PMC3857563

[4] Bakker J. The role of steroid hormones in the sexual differentiation of the human brain. J Neuroendocrinol. 2022. 10.1111/jne.13050. 34708466 10.1111/jne.13050

[5] Balen AH, Conway GS, Kaltsas G, Techatraisak K, Manning PJ, West C, Jacobs HS. Andrology: polycystic ovary syndrome: the spectrum of the disorder in 1741 patients. Hum Reprod. 1995;10(8):2107–11. 10.1093/oxfordjournals.humrep.a136243. 8567849 10.1093/oxfordjournals.humrep.a136243

[6] Bangasser DA, Valentino RJ. Sex differences in stress-related psychiatric disorders: neurobiological perspectives. Front Neuroendocrinol. 2014;35(3):303–19. 10.1016/j.yfrne.2014.03.008. 24726661 10.1016/j.yfrne.2014.03.008 PMC4087049

[7] Bánszegi O, Altbacker V, Bilko A. Intrauterine position influences anatomy and behavior in domestic rabbits. Physiol Behav. 2009;98:258–62. 10.1016/j.physbeh.2009.05.016. 19490922 10.1016/j.physbeh.2009.05.016

[8] Callard IP, Leathem JH. In vitro synthesis of steroids by the adrenal glands of the coypu, Myocastor coypus molina. Acta Endocrinol. 1969;62(4):653–6. 10.1530/acta.0.0620653. 10.1530/acta.0.0620653 5395464

[9] Carter J, Leonard BP. A review of the literature on the worldwide distribution, spread of, and efforts to eradicate the coypu (*Myocastor coypus*). Wildl Soc Bull. 2002;30(1):162–75.

[10] Chippindale AK, Gibson JR, Rice WR. Negative genetic correlation for adult fitness between sexes reveals ontogenetic conflict in Drosophila. Proc Natl Acad Sci. 2001;98(4):1671–5. 10.1073/pnas.98.4.1671. 11172009 10.1073/pnas.98.4.1671 PMC29315

[11] Clutton-Brock TH. Mammalian mating systems. Proc Royal Soc London B Biol Sci. 1989;236(1285):339–72. 10.1098/rspb.1989.0027. 10.1098/rspb.1989.0027 2567517

[12] Cornil CA, Ball GF, Balthazart J. Functional significance of the rapid regulation of brain estrogen action: where do the estrogens come from? Brain Res. 2006;1126(1):2–26. 10.1016/j.brainres.2006.07.098. 16978590 10.1016/j.brainres.2006.07.098 PMC3523229

[13] Critchlow V, Liebelt RA, Bar-Sela M, Mountcastle W, Lipscomb HS. Sex difference in resting pituitary-adrenal function in the rat. Am J Physiol-Legacy Content. 1963;205(5):807–15. 10.1152/ajplegacy.1963.205.5.807. 10.1152/ajplegacy.1963.205.5.807 4291060

[14] Darolti I, Mank JE. Sex-biased gene expression at single-cell resolution: cause and consequence of sexual dimorphism. Evol Lett. 2023;7(3):148–56. 37251587 10.1093/evlett/qrad013 PMC10210449

[15] Di Francesco J, Navarro-Gonzalez N, Wynne-Edwards K, Peacock S, Leclerc LM, Tomaselli M, et al. Qiviut cortisol in muskoxen as a potential tool for informing conservation strategies. Conserv Physiol. 2017;5(1):cox052. 28948023 10.1093/conphys/cox052 PMC5601961

[16] Ellegren H, Parsch J. The evolution of sex-biased genes and sex-biased gene expression. Nat Rev Genet. 2007. 10.1038/nrg2167. 17680007 10.1038/nrg2167

[17] Emery Thompson M, Zhou A, Knott CD. Low testosterone correlates with delayed development in male orangutans. PLoS ONE. 2012;7(10):e47282. 10.1371/journal.pone.0047282. 23077585 10.1371/journal.pone.0047282 PMC3471841

[18] Figueiredo HF, Ulrich-Lai YM, Choi DC, Herman JP. Estrogen potentiates adrenocortical responses to stress in female rats. Am J Physiol-Endocrinol Metab. 2007;292(4):E1173–82. 10.1152/ajpendo.00102.2006. 17179393 10.1152/ajpendo.00102.2006

[19] Fishman R, Vortman Y, Shanas U, Koren L. Female-biased sex ratios are associated with higher maternal testosterone levels in nutria (*Myocastor coypus*). Behav Ecol Sociobiol. 2018;72(6):101. 10.1007/s00265-018-2517-3. 10.1007/s00265-018-2517-3

[20] Fishman R, Vortman Y, Shanas U, Koren L. Cortisol advantage of neighbouring the opposite sex *in utero*. Royal Soc Open Sci. 2018;5(9):171636. 10.1098/rsos.171636. 10.1098/rsos.171636 PMC6170571 30839724

[21] Fishman R, Vortman Y, Shanas U, Koren L. Non-model species deliver a non-model result: nutria female fetuses neighboring males in utero have lower testosterone. Horm Behav. 2019;111:105–9. 10.1016/j.yhbeh.2019.02.011. 30790563 10.1016/j.yhbeh.2019.02.011

[22] Fowden AL, Li J, Forhead AJ. Glucocorticoids and the preparation for life after birth: are there long-term consequences of the life insurance? Proc Nutr Soc. 1998;57(1):113–22. 10.1079/PNS19980017. 9571716 10.1079/PNS19980017

[23] Goel N, Bale TL. Organizational and activational effects of testosterone on masculinization of female physiological and behavioral stress responses. Endocrinology. 2008;149(12):6399–405. 10.1210/en.2008-0433. 18687782 10.1210/en.2008-0433 PMC2613052

[24] Hammes SR, Levin ER. Impact of estrogens in males and androgens in females. J Clin Investig. 2019;129(5):1818–26. 10.1172/JCI125755. 31042159 10.1172/JCI125755 PMC6486327

[25] Handa RJ, Weiser MJ. Gonadal steroid hormones and the hypothalamo–pituitary–adrenal axis. Front Neuroendocrinol. 2014;35(2):197–220. 24246855 10.1016/j.yfrne.2013.11.001 PMC5802971

[26] Heck AL, Handa RJ. Sex differences in the hypothalamic–pituitary–adrenal axis’ response to stress: an important role for gonadal hormones. Neuropsychopharmacology 2019;44(1):45–58. 30111811 10.1038/s41386-018-0167-9 PMC6235871

[27] Hau M. Regulation of male traits by testosterone: implications for the evolution of vertebrate life histories. BioEssays. 2007;29(2):133–44. 10.1002/bies.20524. 17226801 10.1002/bies.20524

[28] Hensch TK. Critical period regulation. Annu Rev Neurosci. 2004;27(1):549–79. 10.1146/annurev.neuro.27.070203.144327. 15217343 10.1146/annurev.neuro.27.070203.144327

[29] Hillman N, Kallapur SG, Jobe A. Physiology of transition from intrauterine to extrauterine life. Clin Perinatol. 2012;39(4):769–83. 10.1016/j.clp.2012.09.009. 23164177 10.1016/j.clp.2012.09.009 PMC3504352

[30] Ingleby FC, Flis I, Morrow EH (2015) Sex-biased gene expression and sexual conflict throughout development. Cold Spring Harbor Perspect Biol. 2015;7(1):a017632. 10.1101/cshperspect.a017632 PMC4292171 25376837

[31] James WH. Hypothesis: high levels of maternal adrenal androgens are a major cause of miscarriage and other forms of reproductive suboptimality. J Theor Biol. 2015;364:316–20. 10.1016/j.jtbi.2014.09.027. 25264266 10.1016/j.jtbi.2014.09.027

[32] Kawata M. Roles of steroid hormones and their receptors in structural organization in the nervous system. Neurosci Res. 1995;24(1):1–46. 10.1016/0168-0102(96)81278-8. 8848287 10.1016/0168-0102(96)81278-8

[33] Kawata M, Yuri K, Ozawa H, Nishi M, Ito T, Hu Z, Lu H, Yoshida M. Steroid hormones and their receptors in the brain. J Steroid Biochem Mol Biol. 1998;65(1):273–80. 10.1016/S0960-0760(98)00026-0. 9699881 10.1016/S0960-0760(98)00026-0

[34] Khalil S, Enbody ED, Frankl-Vilches C, Welklin JF, Koch RE, Toomey MB, Sin SYW, Edwards SV, Gahr M, Schwabl H, Webster MS, Karubian J. Testosterone coordinates gene expression across different tissues to produce carotenoid-based red ornamentation. Mol Biol Evol. 2023;40(4):056. 10.1093/molbev/msad056. 10.1093/molbev/msad056 PMC10072822 36911907

[35] Kishk, W. H. (2008). Interrelationship between ram plasma testosterone level and some semen characteristics. *Slovak Journal of Animal Science*, *41*(2).

[36] Koren L, Geffen E. Androgens and social status in female rock hyraxes. Anim Behav. 2009;77(1):233–8. 10.1016/j.anbehav.2008.09.031. 10.1016/j.anbehav.2008.09.031

[37] Koren L, Bryan H, Matas D, Tinman S, Fahlman A, Whiteside D, Smits J, Wynne-Edwards K. Towards the validation of endogenous steroid testing in wildlife hair. J Appl Ecol. 2019;56(3):547–61. 10.1111/1365-2664.13306. 10.1111/1365-2664.13306

[38] Koren L, Weissman Y, Schnitzer I, Beukeboom R, Bar Ziv E, Demartsev V, Barocas A, Ilany A, Geffen E. Sexually opposite effects of testosterone on mating success in wild rock hyrax. Behav Ecol. 2019;30(6):1611–7. 10.1093/beheco/arz125. 10.1093/beheco/arz125

[39] Lattin CR, Keniston DE, Reed JM, Romero LM. Are receptor concentrations correlated across tissues within individuals? A case study examining glucocorticoid and mineralocorticoid receptor binding. Endocrinology. 2015;156(4):1354–61. 10.1210/en.2014-1811. 25668065 10.1210/en.2014-1811

[40] LeWinn KZ, Stroud LR, Molnar BE, Ware JH, Koenen KC, Buka SL. Elevated maternal cortisol levels during pregnancy are associated with reduced childhood IQ. Int J Epidemiol. 2009;38(6):1700–10. 10.1093/ije/dyp200. 19423658 10.1093/ije/dyp200 PMC2786250

[41] Liebl AL, Shimizu T, Martin LB. Covariation among glucocorticoid regulatory elements varies seasonally in house sparrows. Gen Comp Endocrinol. 2013;183:32–7. 10.1016/j.ygcen.2012.11.021. 23262276 10.1016/j.ygcen.2012.11.021

[42] Liggins G. The role of cortisol in preparing the fetus for birth. Reprod Fertil Dev. 1994;6(2):141. 10.1071/RD9940141. 7991781 10.1071/RD9940141

[43] MacAdams MR. Reduction of serum testosterone levels during chronic glucocorticoid therapy. Ann Intern Med. 1986;104(5):648. 10.7326/0003-4819-104-5-648. 3083749 10.7326/0003-4819-104-5-648

[44] MacManes MD, Austin SH, Lang AS, Booth A, Farrar V, Calisi RM. Widespread patterns of sexually dimorphic gene expression in an avian hypothalamic–pituitary–gonadal (HPG) axis. Sci Rep. 2017;7:45125. 10.1038/srep45125. 28417958 10.1038/srep45125 PMC5394691

[45] Mahfouz A, Lelieveldt BPF, Grefhorst A, van Weert LTCM, Mol IM, Sips HCM, van den Heuvel JK, Datson NA, Visser JA, Reinders MJT, Meijer OC. Genome-wide coexpression of steroid receptors in the mouse brain: Identifying signaling pathways and functionally coordinated regions. Proc Natl Acad Sci. 2016;113(10):2738–43. 10.1073/pnas.1520376113. 26811448 10.1073/pnas.1520376113 PMC4791033

[46] Marciniak B, Patro-Ma J. Glucocorticoids in pregnancy. Curr Pharm Biotechnol. 2011;12(5):750–7. 21342122 10.2174/138920111795470868

[47] Matas D, Doniger T, Sarid S, Asfur M, Yadid G, Khokhlova IS, Krasnov BR, Kam M, Degen AA, Koren L. Sex differences in testosterone reactivity and sensitivity in a non-model gerbil. Gen Comp Endocrinol. 2020;291:113418. 10.1016/j.ygcen.2020.113418. 32027878 10.1016/j.ygcen.2020.113418

[48] McEwen BS. Protective and damaging effects of stress mediators. N Engl J Med. 1998;338:171–9. 10.1056/NEJM199801153380307. 9428819 10.1056/NEJM199801153380307

[49] McGlothlin JW, Jawor JM, Greives TJ, Casto JM, Phillips JL, Ketterson ED. Hormones and honest signals: males with larger ornaments elevate testosterone more when challenged. J Evol Biol. 2008;21(1):39–48. 10.1111/j.1420-9101.2007.01471.x. 18034801 10.1111/j.1420-9101.2007.01471.x

[50] Meitzen J, Meisel RL, Mermelstein PG. Sex differences and the effects of estradiol on striatal function. Curr Opin Behav Sci. 2018;23:42–8. 10.1016/j.cobeha.2018.03.007. 30221186 10.1016/j.cobeha.2018.03.007 PMC6136839

[51] Mills S, Grapputo A, Jokinen I, Koskela E, Mappes T, Oksanen T, Poikonen T. Testosterone-mediated effects on fitness-related phenotypic traits and fitness. Am Nat. 2009;173:475–87. 10.1086/597222. 19236274 10.1086/597222

[52] Moisiadis VG, Matthews SG. Glucocorticoids and fetal programming part 1: outcomes. Nat Rev Endocrinol. 2014. 10.1038/nrendo.2014.73. 24863382 10.1038/nrendo.2014.73

[53] Morton S, Brodsky D. Fetal physiology and the transition to extrauterine life. Clin Perinatol. 2016;43(3):395–407. 10.1016/j.clp.2016.04.001. 27524443 10.1016/j.clp.2016.04.001 PMC4987541

[54] Muff S, Nilsen EB, O’Hara RB, Nater CR. Rewriting results sections in the language of evidence. Trends Ecol Evol. 2022;37(3):203–10. 10.1016/j.tree.2021.10.009. 34799145 10.1016/j.tree.2021.10.009

[55] Newson RM (1966) Reproduction in the feral coypu (*Myocastor coypus*). In: Symposia of the Zoological Society of London, vol. 15. p. 323–334.

[56] Ng PC. The fetal and neonatal hypothalamic-pituitary-adrenal axis. Arch Dis Child - Fetal Neonatal Ed. 2000;82(3):250F – 254. 10.1136/fn.82.3.F250. 10.1136/fn.82.3.F250 PMC1721090 10794797

[57] Ochedalski T, Subburaju S, Wynn PC, Aguilera G. Interaction between oestrogen and oxytocin on hypothalamic-pituitary-adrenal axis activity. J Neuroendocrinol. 2007;19(3):189–97. 10.1111/j.1365-2826.2006.01525.x. 17280592 10.1111/j.1365-2826.2006.01525.x

[58] Panagiotakopoulos L, Neigh GN. Development of the HPA axis: Where and when do sex differences manifest? Front Neuroendocrinol. 2014;35(3):285–302. 10.1016/j.yfrne.2014.03.002. 24631756 10.1016/j.yfrne.2014.03.002

[59] Rice D, Barone S Jr. Critical periods of vulnerability for the developing nervous system: evidence from humans and animal models. Environ Health Perspect. 2000;108:23. 10852851 10.1289/ehp.00108s3511 PMC1637807

[60] Ronald A, Pennell C, Whitehouse A. Prenatal maternal stress associated with ADHD and autistic traits in early childhood. Front Psychol. 2011. 10.3389/fpsyg.2010.00223. 21833278 10.3389/fpsyg.2010.00223 PMC3153828

[61] Rosvall KA, Bergeon Burns CM, Barske J, Goodson JL, Schlinger BA, Sengelaub DR, Ketterson ED. Neural sensitivity to sex steroids predicts individual differences in aggression: implications for behavioural evolution. Proc Royal Soc B: Biol Sci. 2012;279(1742):3547–55. 10.1098/rspb.2012.0442. 10.1098/rspb.2012.0442 PMC3396890 22673360

[62] Rosvall KA, Bergeon Burns CM, Hahn TP, Ketterson ED. Sources of variation in HPG axis reactivity and individually consistent elevation of sex steroids in a female songbird. Gen Comp Endocrinol. 2013;194:230–9. 10.1016/j.ygcen.2013.09.015. 24090613 10.1016/j.ygcen.2013.09.015 PMC3852689

[63] Ryan BC, Vandenbergh JG. Intrauterine position effects. Neurosci Biobehav Rev. 2002;26(6):665–78. 10.1016/S0149-7634(02)00038-6. 12479841 10.1016/S0149-7634(02)00038-6

[64] Ryan CP, Anderson WG, Gardiner LE, Hare JF. Stress-induced sex ratios in ground squirrels: support for a mechanistic hypothesis. Behav Ecol. 2012;23(1):160–7. 10.1093/beheco/arr169. 10.1093/beheco/arr169

[65] Schöpper H, Klaus T, Palme R, Ruf T, Huber S. Sex-specific impact of prenatal stress on growth and reproductive parameters of guinea pigs. J Comp Physiol B. 2012;182(8):1117–27. 10.1007/s00360-012-0680-9. 22714716 10.1007/s00360-012-0680-9

[66] Seale JV, Wood SA, Atkinson HC, Harbuz MS, Lightman SL. Gonadal steroid replacement reverses gonadectomy-induced changes in the corticosterone pulse profile and stress-induced hypothalamic-pituitary-adrenal axis activity of male and female rats. J Neuroendocrinol. 2004;16(12):989–98. 10.1111/j.1365-2826.2004.01258.x. 15667454 10.1111/j.1365-2826.2004.01258.x

[67] Sherry DF, Galef J, Bennett G, Clark MM. Sex and intrauterine position influence the size of the gerbil hippocampus. Physiol Behav. 1996;60(6):1491–4. 10.1016/S0031-9384(96)00311-3. 8946496 10.1016/S0031-9384(96)00311-3

[68] Simerly RB, Swanson LW, Chang C, Muramatsu M. Distribution of androgen and estrogen receptor mRNA-containing cells in the rat brain: an in situ hybridization study. J Comp Neurol. 1990;294(1):76–95. 10.1002/cne.902940107. 2324335 10.1002/cne.902940107

[69] Smith KD, Rodriguez-Rigau LJ, Tcholakian RK, Steinberger E. The relation between plasma testosterone levels and the lengths of phases of the menstrual cycle. Fertil Steril. 1979;32(4):403–7. 10.1016/S0015-0282(16)44295-0. 488426 10.1016/S0015-0282(16)44295-0

[70] Staub NL, De Beer M. The role of androgens in female vertebrates. Gen Comp Endocrinol. 1997;108(1):1–24. 10.1006/gcen.1997.6962. 9378263 10.1006/gcen.1997.6962

[71] Surbek D, Drack G, Irion O, Nelle M, Huang D, Hoesli I. Antenatal corticosteroids for fetal lung maturation in threatened preterm delivery: indications and administration. Arch Gynecol Obstet. 2012;286(2):277–81. 10.1007/s00404-012-2339-x. 22543752 10.1007/s00404-012-2339-x

[72] Thompson ME, Zhou A, Knott CD. Low testosterone correlates with delayed development in male orangutans. PLoS ONE. 2012;7(10):e47282. 10.1371/journal.pone.0047282. 23077585 10.1371/journal.pone.0047282 PMC3471841

[73] Tobiansky DJ, Wallin-Miller KG, Floresco SB, Wood RI, Soma KK. Androgen regulation of the mesocorticolimbic system and executive function. Front Endocrinol. 2018. 10.3389/fendo.2018.00279. 10.3389/fendo.2018.00279 PMC5996102 29922228

[74] Uhart M, Chong R, Oswald L, Lin P-I, Wand G. Gender differences in hypothalamic-pituitary-adrenal (HPA) axis reactivity. Psychoneuroendocrinology. 2006;31:642–52. 10.1016/j.psyneuen.2006.02.003. 16616815 10.1016/j.psyneuen.2006.02.003

[75] Voigt C, Goymann W. Sex-role reversal is reflected in the brain of African black coucals (*Centropus grillii*). Dev Neurobiol. 2007;67(12):1560–73. 10.1002/dneu.20528. 17542014 10.1002/dneu.20528

[76] vom Saal FS. Variation in phenotype due to random intrauterine positioning of male and female fetuses in rodents. Reproduction. 1981;62(2):633–50. 10.1530/jrf.0.0620633. 10.1530/jrf.0.0620633 7252935

[77] vom Saal FS, Bronson FH. Sexual characteristics of adult female mice are correlated with their blood testosterone levels during prenatal development. Science. 1980;208(4444):597–9. 10.1126/science.7367881. 7367881 10.1126/science.7367881

[78] Weinstock M. The potential influence of maternal stress hormones on development and mental health of the offspring. Brain Behav Immun. 2005;19(4):296–308. 10.1016/j.bbi.2004.09.006. 15944068 10.1016/j.bbi.2004.09.006

[79] Wolf CJ, Hotchkiss A, Ostby JS, LeBlanc GA, Gray LE Jr. Effects of prenatal testosterone propionate on the sexual development of male and female rats: a dose-response study. Toxicol Sci. 2002;65(1):71–86. 10.1093/toxsci/65.1.71. 11752687 10.1093/toxsci/65.1.71

[80] Zachry JE, Nolan SO, Brady LJ, Kelly SJ, Siciliano CA, Calipari ES. Sex differences in dopamine release regulation in the striatum. Neuropsychopharmacology. 2021. 10.1038/s41386-020-00915-1. 33318634 10.1038/s41386-020-00915-1 PMC8027008

[81] Zambrano E, Guzman C, Rodríguez-González G, Durand-Carbajal M, Nathanielsz P. Fetal programming of sexual development and reproductive function. Mol Cell Endocrinol. 2013. 10.1016/j.mce.2013.09.008. 24045010 10.1016/j.mce.2013.09.008

[82] Zohar I, Weinstock M. Differential effect of prenatal stress on the expression of cortiocotrophin-releasing hormone and its receptors in the hypothalamus and amygdala in male and female rats. J Neuroendocrinol. 2011;23(4):320–8. 10.1111/j.1365-2826.2011.02117.x. 21306450 10.1111/j.1365-2826.2011.02117.x

