# Supplementary material for: Fetal endocrine axes mRNA expression levels are related to sex and intrauterine position
Source: Biol Sex Differ. 2024 Aug 5;15:61. doi: 10.1186/s13293-024-00637-9 (PMC11301978; doi:10.1186/s13293-024-00637-9)
Supplement: Supplementary file 1 — Supplementary Material 1. [file 13293_2024_637_MOESM1_ESM.pdf]

Supplementary Information (SI)

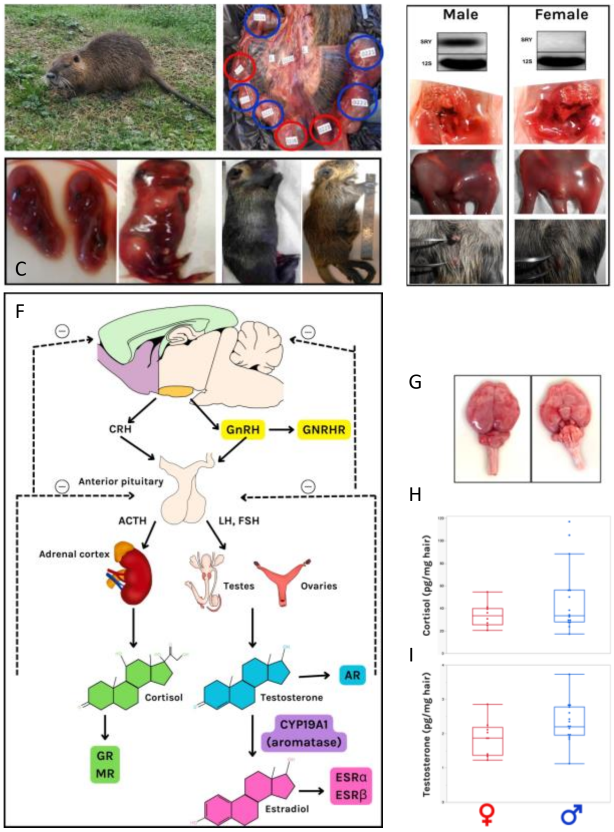

Figure S1. The nutria study system. A: An adult nutria. B: Uterine horns of a female nutria with 8 fetuses (5 males (circled blue) and 3 females (circled red)) spread over the left and right horns. C: Developmental stages of nutria fetuses. From left to right, nutria fetuses at 58-, 90-, 115- and 133-days gestation. Hair-testing is possible from ~110 days of gestation. Prior to ~80-90 days gestation, brain areas are not visibly distinguishable and are not collected. D & E: Fetal sex based on PCR (necessary at < 77 days of gestation; showing SRY (only in males) vs. a housekeeping gene present in both sexes), internal anatomy (shown at ~ 80 days gestation), or external anatomy (via AGD; possible at > 90 days of gestation). Ano-genital distance (AGD) is longer in males than in females due to *in utero* T. F: HPA and HPG axes, highlighting the brain areas that were sampled (prefrontal cortex in green, striatum in purple, and hypothalamus in orange) and the axes' components whose expression levels were quantified (GnRH, GNRHR, AR, aromatase, ESR1, GR, and MR). G: Ventral (left) and dorsal (right) view of the well-developed fetal nutria brain. H: No significant differences in hair cortisol levels between male (blue) and female (red) nutria fetuses (t $\text{ratio}_{19.143} = 1.66$, P = 0.057). I: Hair testosterone levels are higher in male nutria fetuses (blue) than in female nutria fetuses (red; t $\text{ratio}_{18.949} = 1.903$; P = 0.036).

Table 1A. Exon structure for HPA and HPG axes components in *Myocastor coypus* genome

**Gene**	**Myocastor coypus (MyoCoy_v1_BIUU)**
**Androgen receptor (AR)**	Exon 1: PVJA010042438.1: 8921-10498 (-)<br>Exon 2: PVJA010007205.1: 68316-68197 (-)<br>Exon 3: PVJA010007205.1: 45473-45174 (-)<br>Exon 4: PVJA010007205.1: 40157-40011 (-)<br>Exon 5: PVJA010007205.1: 36122-35991 (-)<br>Exon 6: PVJA010007205.1: 35134-34967 (-)<br>Exon 7: PVJA010007205.1: 34298-34077 (-)<br>Exon 8: PVJA010019829.1: 13434-13276 (-)
**Gonadotropin-releasinghormone (GNRH)**	Exon 1: PVJA010000540.1: 26875-27027 (+)<br>Exon 2: PVJA010000540.1: 28210-28308 (+)<br>Exon 3: PVJA010000540.1: 29407-29448 (+)
**Gonadotropin-releasing hormone receptor (GNRHR)**	Exon 1: PVJA010011111.1: 16738-17247 (-)<br>Exon 2: PVJA010011111.1: 5411-5630 (-)<br>Exon 3: PVJA010011111.1: 4346-4590 (-)
**Estrogen receptor α (ESR1)**	Exon 1: PVJA010009086.1: 15958-16452 (+)<br>Exon 2: PVJA010009086.1 : 46816-47022 (+)<br>Exon 3: PVJA010012292.1: 48810-48911(-)<br>Exon 4: PVJA010048306.1: 8155-8490 (-)<br>Exon 5: PVJA010011844.1: 6604-6822(+)

	Exon 6: PVJA010011844.1: 39894-40058(+)<br>Exon 7: PVJA010040007.1: 15935- 16123(+)<br>Exon 8: PVJA010071099.1: 4598-4831(-)
**Aromatase (CYP19A1)**	Exon 1: PVJA010009970.1: 13692-13836(+)<br>Exon 2: PVJA010009970.1: 18215-18365(+)<br>Exon 3: PVJA010009970.1: 25544-25698(+)<br>Exon 4: PVJA010009970.1: 29630-29806(+)<br>Exon 5: PVJA010009970.1: 32598-32712(+)<br>Exon 6: PVJA010009970.1: 36760-36874(+)<br>Exon 7: PVJA010009970.1: 37887-38049(+)<br>Exon 8: PVJA010009970.1: 40027-40268(+)<br>Exon 9: PVJA010009970.1: 44238-44486(+)
**Glucocorticoid receptor (GR)**	Exon 1: PVJA010007953.1: 3668-2493(-)<br>Exon 2: PVJA010003780.1: 59033-58860(-)<br>Exon 3: PVJA010003780.1: 55938-55819(-)<br>Exon 4: PVJA010003780.1: 53003-52725(-)<br>Exon 5: PVJA010003780.1: 51914-51768(-)<br>Exon 6: PVJA010003780.1: 45639-45496(-)<br>Exon 7: PVJA010003780.1: 43861-43700(-)<br>Exon 8: PVJA010003780.1: 43219-43067(-)
**Mineralocorticoid receptor (MR)**	Exon 1: PVJA010024645.1: 21418-23163(+)<br>Exon 2: PVJA010032199.1: 3382-3528(+)<br>Exon 3: PVJA010008420.1: 49401-49520(+)<br>Exon 4: PVJA010035199.1: 6540-6848(-)<br>Exon 5: PVJA010035199.1: 4932-5078(-)<br>Exon 6: PVJA010001201.1: 19749-19907(+)<br>Exon 7: PVJA010001201.1: 25596-25766(+)<br>Exon 8: PVJA010001201.1: 55389-55541(+)
**Glycogen synthase 1 (Gys1)**	Exon 1: PVJA010041420.1: 9867-9990(+)<br>Exon 2: PVJA010041420.1: 11315-11516(+)<br>Exon 3: PVJA010041420.1: 12950-13210(+)<br>Exon 4: PVJA010041420.1: 14856-15068(+)<br>Exon 5: PVJA010041420.1: 15203-15360 (+)<br>Exon 6: PVJA010010477.1: 12145-12279(+)<br>Exon 7: PVJA010010477.1: 12437-12568(+)<br>Exon 8: PVJA010010477.1: 15391-15501(+)<br>Exon 9: PVJA010010477.1: 16092-16172(+)<br>Exon 10: PVJA010010477.1: 16728-16823(+)<br>Exon 11: PVJA010010477.1: 18268-18387(+)<br>Exon 12: PVJA010010477.1: 28043-28168(+)<br>Exon 13: PVJA010075489.1: 2682-2780(-)<br>Exon 14: PVJA010075489.1: 2292-2531(-)<br>Exon 15: PVJA010075489.1: 1894-1992(-)<br>Exon 16: PVJA010075489.1: 1430-1765(-)

Table 1B. Coding Sequences for HPA and HPG axes components in *Myocastor coypus* genome

Gene	Myocastor coypus (MyoCoy_v1_BIUU)
**Androgen receptor (AR)**	ATGGAAGTGCAGTTGGGGCTGGGGAGGGTCTACCCCCGGCCGCCGTCCAAGACCTTT<br>CGAGGAGCTTTCCAGAATTTGTTCCAAAGCGTGCGCGAAGTCATCCAGACCCCAGGG<br>TCCCGCCATCCTGAGGCCGATAGCACAGCACCTCTAGGCGCCCATGTGCAGCAGCAG<br>GAGACCAGCGCCCGGCAGCCGCATCAGGGTGAGGATGGCTCTCCCCAAGCTCACAT<br>CAGAGGCCCCACAGGCTACCTGGCCCTGGAGGAGGAACAGCAGCCTTCACAACAGC<br>AGCCAACCTCTGAGGGCCACCCTGAGAGGGGCTGTCTCTCAGAGCCTGGAACCTCTG<br>TGCCCGCCAGCAAGGGGTTGCCGCAGCAGCCAGCACCTCCGGACGAGAATGACTCA<br>GCTGCCCCATCCACGCTGTCTCTGCTGGGCCCCACTTTCTCCAGCTTAACCAGCTGCT<br>CCACCGATCTTAAAGACCTACTGAGTGAAGCCAACAGCATGCAGCTTCTTCAGCAGC<br>CACAGCAGCAGCCGCAGCCACAGCAGCCTCAGCCTCAGCCCCAGCCCCAGCCGCAG

	CAGCCACCGCAGCAGCAGCAGCCACAGCAGCTAGAAGTGGTATCTGAAACCACCAG<br>CAGCGGCAGACTAAGAGAGGGAGTTGGGGCTGCCACCTCTTCCAAGGACAGTTACC<br>TAGGGGTCAATTCCACCATATCTGACAGCGCCAAGGAGTTGTGTAAGGCAGTGTCTG<br>TGTCTATGGGCTTGGGTGTGGAGGCACTGGAGCATCTGAGCCCTGGGGAACAGCTTC<br>GGGGAGACTGCATGTATGCTCCACTTCTGGGAGGTCCACCTGCCATGCGACAAACTC<br>ACTGTGCCCCACTGGCAGAGTGCAAAGGTTCTCTGCTGAATGACAGCGCAGGCAAA<br>GGCGCTGATGAGACTGTGGACTATTCCTCTTTCAAGGGAGGTTACTCCAAAGGACTA<br>GACAGCGAAAGCTTGGGCTGCTCTGGCAGCAGCGAAGCAGGGAGCTCTGGGACACT<br>TGAATTGCCCTCCACCCTGTCTCTCTACAAGTCTGGAGCAGTGGAGGAGGAAGCATC<br>ATACCAGAGTCGGGACTACTACAACTTTCCGCTGCCCCTGGCCGGGCCTCCACCCCC<br>TCCACAACCTCCCCATCCACACACCCGCATCAAGCTGGAGAACCCGCTGGACTATAG<br>CAGTGCCTGGGCAGCCGCATCGGCACAGTGCCGCTATGGAGACCTGGCAAGCCTGC<br>ATGGTGGGGGTGTCCCAGGACCCAGCTCTGGGTCACCCTCAGCCACTGCCTCATCTT<br>CCTGGCATACTCTTTTCACAGCGGAAGAAGGCCAGTTGTATGGACCGTGTGGAGGGA<br>GTGGCGGAGGCAGCTCCGGAGAGGCAGGCTCTGTAGCCCCCTATGGTTATGCTCGGC<br>CACCACAGGGTCTTGCAGGTCAGGAAGGTGATTTCCCTGCAACCGATGTATGGTACC<br>CCGGTGGCATGGCAAGTAGAGTGCCCTATCACAGTCCAGGTTGTGTCAAAAGCGAG<br>ATGGGACCCTGGATGGAGAGCTACTCTGGACCTTATGGAGACATGCGTTTGGAGAAT<br>GCCAGGGACCACGTTTTGCCCATCGACTATTACTTTCCTCCCCAGAAGACCTGCCTG<br>ATCTGTGGTGATGAAGCTTCTGGGTGTCACTATGGAGCTCTCACTTGTGGCAGCTGC<br>AAGGTCTTCTTCAAAAGAGCTGCTGAAGGAAAACAGAAGTACTTGTGTGCCAGTAG<br>AAATGATTGCACCATTGATAAATTCCGAAGGAAAAATTGTCCATCTTGTCGTCTTCG<br>AAAATGCTATGAAGCAGGAATGACTCTGGGAGCCCGGAAGCTGAAGAAACTTGGCA<br>ATTTAAAACTACAGGAAGAATCAGAAACTGCCAGTGCCAGCAGCCCCACTGAAGAG<br>CCATCTCAGAAGATGTCAGTATCACATATTGAAGGCTATGACTGTCAGCCCATCTTC<br>CTAAATGTCCTAGAAGCCATTGAGCCAGGTGTGGTGTGTGCTGGACATGACAACAAC<br>CAACCTGACTCCTTTGCAACCTTGCTGTCTAGCCTCAATGAACTAGGGGAGAGACAG<br>CTTGTACATGTGGTGAAGTGGGCCAAAGCCTTGCCTGGCTTCCGCAACTTGCATGTG<br>GATGACCAGATGGCAGTCATTCAGTACTCCTGGATGGGACTCATGGTATTTGCCATG<br>GGCTGGCGGTCCTTCACCAATGTCAACTCCAGAATGCTCTACTTTGCACCTGACCTA<br>GTTTTCAATGAGTATCGCATGCATAAGTCCCGGATGTACAGCCAATGCGTCCGAATG<br>AGGCATCTCTCGCAAGAGTTTGGATGGCTCCAAATCACACCCCAGGAGTTCCTTTGC<br>ATGAAAGCACTGCTACTCTTCAGCATTATTCCAGTGGATGGGCTGAAAAATCAAAAA<br>TTCTTTGATGAACTTCGAATGAACTACATCAAGGAACTCGATCGTATCATTGCATGC<br>AAAAGAAAAAATCCCACATCCTGCTCAAGGCGCTTCTACCAGCTCACAAAGCTCCTG<br>GACTCTGTGCAGCCTATTGCAAGAGAGCTGCATCAGTTCACTTTTGACCTGCTAATC<br>AAGTCACACATGGTGAGCGTGGACTTCCCGGAAATGATGGCAGAGATCATCTCTGTG<br>CAAGTGCCCAAGATCCTTTCTGGGAAAGTCAAGCCCATCTATTTCCATACACAGTGA
**Gonadotropin-releasing hormone (GNRH)**	ATGCTTCTTAGAATGGGGCCAGTTCCCAGACTCCTGGCTGGACTTGTCCTGTTGACTT<br>TATGTGTGGAACATGGCTCAGGCCAACACTGGTCCTATGGCCTGCGTCCTGGAGGAA<br>AGAGAAATGCTGAAACCCTGGTTGAGTCTTTCCAAGAGATAGCCCAAGAGATCGAT<br>CAACTGGCGGAACCCCAGCAGCATCTCGAATGTACCCTCCACCGGCCTCACTCTCCC<br>CTCAGGGACCTGAAAGGAGCTCTGGAAAGTCTGATTGAAGAGGAAACCGGTCAGAA<br>AAAGATTTAA
**Gonadotropin-releasing hormone receptor (GNRHR)**	ATGGAAAACAGTGCCTCTTCTGAACAGAATCAAAACCTTTGCAACAACAGTGTCCCA<br>TTGATGCAGGGGACTCTTCCCACTCTGACTTTATCTGGAAAGATCCGAGTGACAGTG<br>ACTTTGGTCCTTTTTCTACTCTCCACCACCTTTAATGCTTCTTTCTTATTGAAACTTAG<br>GAAATGGACTCAGAAGGAGAAAGGAAAAAAGCTCTCAAGAATGAAAGTGCTTTTGA<br>AACACCTAACTTTAGCCAACCTGTTGGAGACTCTGATTGTCATGCCCCTGGATGGGC<br>TGTGGAACATTACAGTCCAATGGTATGCAGGAGAGTTACTCTGTAAAGTCCTCAGCT<br>ATCTGAAGCTTTTCTCCATGTATGCCCCTGCCTTCATGATGGTGGTAATCAGCCTGGA<br>CCGCTCTCTAGCCATCACCAGGCCCCTACCTGTGCAAAGCAGCCAGAAGCTTGAACA<br>GTCCATGATTGGCCTGGCCTGGGTCCTCAGTAGTGCCTTTGCTGGGCCACAGTTATAT<br>ATCTTCAAGATGATCTACCTCCAAAATGGTTCTGGACAAACAGAAGTTTTCTCCCAG<br>TGTGTAACACACTGTAGCTTTCCACAGTGGTGGCATCAGGCTTTCTATAACCTCTTCA<br>CCTTCAGCTGCCTGTTTATTATCCCTCTTCTCATCATGCTGATCTGCAATGCAAAAAT<br>CATCTTTACTCTGACACAGGTCCTTCAGCAGGATCCGCACAAACTACAACTCAATCA<br>ATCCAAGAACAATATACCAAGAGCTCGGCTGAGGACACTGAAGATGACAGTGGCGT<br>TTGCCTCTTCCTTTATTATCTGCTGGACTCCCTACTATGTCCTAGGAATTTGGTATTGG<br>TTTGATCCTGGAATGCTAAACAGGGTGTCAGACCCAGTAAATCACTTCTTCTTTCTGT<br>TTGCTTTTTTAAACCCATGCTTTGATCCACTTATATACGGATATTTTTCTCTGTGA
**Estrogen receptor α (ESR1)**	ATGACCATGACCCTCCACACCAAAGCCTCCAGCATGGCCCTGCTGCACCAGATTCAG<br>GCGAGCGAGCTGGAGCCCCTGAGCCGCCCGCAGCTCAAGATGCCCCTGGAGGAGCC<br>GCAGGGCGAGGTGTTCCTGGACGGCGGCAAGCCGTCGGTCTTCAACTACCCCGAGG<br>GTACCGCCTACGAGTTCAATGCTGCGGCCGCCACCTCCGCGCCGGTCTATGGCCAGC<br>CGGGCCTGGCCTACGGCCCCGGGTCCGAGGCAGCCGCGGCGTTCGGGCCCAGCGGC<br>CTGGGGGGCTTCTCGCACCTCAACAGCGTGTCCCCCAGCCCGCTGATGCTGCTGCAC<br>CAGCCGCCGCAGCTGTCGCCCTTCCTACACCCGCACAGCCAGCAGGTGCCCTTCTAC<br>CTGGAGAACGAGCCGAGCGGCTACGCGGTGCGCGAGGCCGGCGCCCCGGCCTTCTA

	CAGGCCAAATTCAGGTAATCGACGCCAGAATGGCCGAGAGAGATTGGCCAGCAGCA<br>GCGACAAGGGAAGCATAGCCCTGGAGTCTGCCAAGGAGACCCGCTACTGCGCAGTG<br>TGCAATGACTATGCCTCTGGCTACCATTATGGCGTCTGGTCCTGTGAGGGCTGCAAG<br>GCTTTCTTCAAGAGAAGTATTCAAGGGCATAACGACTACATATGTCCAGCTACCAAC<br>CAGTGCACGATTGATAAAAACAGGAGGAAGAGCTGTCAGGCCTGCCGGCTTCGCAA<br>GTGCTATGACGTAGGCATGATCAAAGGTGGGATACGGAAAGACCGAAGAGGAGGG<br>AGAATGTTGAAATATAAGCGACAAAGAGATGATGAAGACAGAAGGAATGAGATGG<br>GGCCCTCTGCAGACATGAGAGCTTCTAACCTTTGGCCAAGCCCTCTTGTAATTAAGC<br>ACACGAAGAAAAACAGCCCAGCCTTGTCCTTGACAGCGGATCAGATGGTCAGTGCC<br>TTGATGGAGGCCGAGCCACCCTTAATCTATTCTGAGTATGATTCTACCAAACCCTTCA<br>GTGAAGCTTCAATGATGGGCTTATTGACCAACCTGGCAGATAGGGAGCTGGTGCATA<br>TGATCAACTGGGCAAAGAGGGTACCAGGTTTTGGGGATTTGAACCTCCATGACCAGG<br>TTCACCTTTTAGAATGTGCCTGGCTAGAGATCCTCATGATTGGACTTGTCTGGCGCTC<br>CATGGAGCACCCTGGGAAGCTCCTGTTTGCTCCTAACTTGATCTTGGACAGGAATCA<br>AGGTGAATGTGTAGAGGGCATGGTGGAGATCTTTGACATGTTGCTGGCGACATCAGC<br>TCGGTTCCGTATGATGAATCTGCAGGGAGAGGAGTTTGTGTGCCTCAAATCTATCAT<br>TTTGCTTAATTCTGGAGTGTACACCTTTCTGTCCAGCACCTTGAAGTCTCTGGAAGAG<br>AAGGACCATATCCACCGGGTCCTGGACAAGATCACAGACACCTTGATCCACCTGATG<br>GCCAAAGCAGGCTTGACTCTGCAGCAGCAGCACCGGCGCCTGGCTCAGCTCCTCCTC<br>ATCCTCTCCCACTTTCGGCACATGAGTAACAAAGGCATGGAACATCTGTACAACATG<br>AAGTGCAAGAACGTGGTGCCCCTGTATGACCTGCTGCTGGAGATGCTGGAGGCCCAT<br>CGCCTGAACACGCCCTCCAACCCCATGGGCGGGTCGCCGGAGGAGCCCAGCCAGAG<br>CCAGCTGGCCATGATGGGCTCTTCTTCAGCACATTCCCTGCAACCATACTATGTCCCA<br>CAGGAAGCGGAGAGTTTCCCCAACACAGTTTGA
**Aromatase (CYP19A1)**	ATGGCCCTGGAAATGCTGAACCCAATGAACCCTAACATCACCAGCATGGTGCCTGAA<br>GTCGTGCCCGCTGCTGCCATGCCAATCCTGCTGCTTATGGGCTTCATCCTTTTGATTT<br>GGAACTGTGAGAGTACTTCCTCAATTCCAGGTCCTGGCTACTGTCTGGGAATTGGGC<br>CGCTCATTTCCCATGGCAGATTCCTGTGGATGGGGATTGGCAGAGCCTGCAACTACT<br>ACAACAAGACATATGGAGAATTCATGAGAGTCTGGATAAATGGAGAGGAAACACTT<br>ATCATCAGCAAGTCCTCAGGTATGTTCCATGTAATGAAGCACACTCACTATGTATCC<br>CGATTTGGCAGCAAGCCCGGGCTACAGTGCATTGGGATGCACGAGAACGGCATTAT<br>ATTTAACAATAACCCAGACATCTGGAAAACTGTTAGACCTTTCTTCATGAAAGCTCT<br>GACAGGCCCTGGCCTTGTTCGCATGGTGGCAATCTGTGCTCAGTCCATCATAAGGCA<br>CCTGGACAGAATGGAGGAGGTCACCAGTGCATCAGGCAGCGTGGACGTGCTCACCC<br>TTATGCGACGCACCATGCTGGACACCTCTAACATACTCTTCCTGGGGATCCCCTTGG<br>ACGAAATTTCCATTGTGAGGAAAATCCAGGCTTATTTTGATGCATGGCAAGCTCTCA<br>TTATAAAACCAAACATCTTCTTTAAGATTTCTTGGCTCTACAAAAAGTATGAAAAGC<br>CTGTGAAGGACTTGAAAGATGCCATAGATAATTTGGTAGAAATAAAAAGACACAAG<br>GTTTGCACAGCAGAGAAACTGGAAGAGTGCATGGATTTTGCCACTGACTTGATTTTT<br>GCTGAGAAACGTGGGGAACTCACAAGGGAGAATGTGAACCAGTGCATCCTGGAGAT<br>GCTCATTGCAGCGCCAGACACCATGTCTGTCTCGGTGTACTTCATGCTGCTGCTTATT<br>GCAGAACACCACAAGGTTGAAGAGGCCATCATGAAGGAAATCCAGACTGTCATTGG<br>TGAACGAGACATAGGAATTGATGATATGCAGAAATTAAAAGTGCTAGAAAACTTCA<br>TCTATGAGAGCATGCGCTACCAGCCTGTGGTGGATTTGGTCATGCGCAAAGCTTTAG<br>AAGATGATGTCATTGATGGCTATACAGTGAAAAAGGGAACTAACATTATCCTAAATA<br>TTGGAAGAATGCATAGACTGGAGTTTTTCCCCAAGCCCGATGAATTTACTCTAGAAA<br>ACTTTGAGAAGAATGTTCCTTACAGGTATTTTCAGCCATTTGGTTTTGGGCCCCGTAG<br>CTGTGCAGGGAAATACATTGCCATGGTGATGATGAAGGTCATCCTGGTGACACTTCT<br>GAGACGATTCCATGTGAAGACACTACAAGAAAGGTGTGTGGAAAGCATACAGAAAA<br>CAAGCAACTTGTCCACACACCCAAATGAGACAGTTGACCTGCCGAAAATGATTTTAA<br>TCCCAAGAAATTCAGAAAAGTGCCTCAAACACTGA
**Glucocorticoid receptor (GR)**	ATGGACCCCAAAGAATCCTTAACCCCTTCCAGTAAAGAAGTCTCCAGCAATGTGCTT<br>GGTTCAGAGAGGAGGAAAGCGATCGATTTCTATAAAACCCTAAGGGGAGGAGCTAC<br>CGTGAATGTTTCTGCATCTTCTCCCTCACTGGCTGCTGCCGCTCAGTCAGATTCCAAG<br>CAGCGAAGACTTCTGGTTGATTTTCCAAAAGGTTCAGGAAGCAATGCACAGCAGCCA<br>GATCTGTCCAAAGCTGTTTCACTGTCAATGGGACTGTATATGGGAGAGACAGAAACA<br>AAAGTGATGGGAAATGACTTGGGATTTCCACAGCAGGGCCAAATCAGCCTTTCCTCT<br>GGAGAAACAGACTTTCAGCTCCTGGAAGAAAGTATTGCAAACCTCAGTAGGTCGAC<br>CAGTATTTCAGAAAACCCTGTGAGTTCAGCATCTGCTGTGTCTGGAACCCTAACAGA<br>GGAGCTTCCCCAAACTCAGTCTGATGTATCTTCAGAACAGCAACACCTGAAGGGCCA<br>GACTGGCACCAATGGTAGCAATGTGAAGTTATATCCCACAGACCAAAGCACCTTTGA<br>CATCTTGCAGGATTTGGAGTTTTCTTCTGGGTCCCCAGGTAAAGAAATGAATGAGAG<br>TCCTTGGAGACCAGACCTGTTGATGGACGAAAACAGTTTGCTTTCTCCTTTGGCAGG<br>AGAGGATGATCCATTCCTTTTGGAAGGAAACACAAGTGAAGACTGCAAGCCTCTTAT<br>TTTACCAGACACTAAACCTAAAATTAAAGATAATGGAGATGTGATCTTATCAAGCCC<br>TAACAGTGTGCCACTGCCCCAAGTCAAAACAGAAAAAGAAGATTTCATTGAACTCTG<br>CACCCCTGGGGTAATTAAGCAGGAGAAACTGGGCCCAGTTTACTGTCAGGCAAGCTT<br>TTCTGGGGCAAATATAATTGGTAATAAAATGTCTGCGATTTCTGTACATGGTGTGAG<br>TACCTCTGGAGGACAGGTTTACCACTATGACATGAATACAGCATCCCTTTCTCAGCA

	GCAGGATCAGAAGCCTATTTTTAATGTCATTCCACCAATTCCTGTTGGTTCAGAAAAT<br>TGGAGTAGGTGCCAAGGATCTGGAGATGACAGCCTGACTTCCCTGGGATCTATGAAC<br>TTCTCTGGTCGATCAGTTTTTTCTAATGGCTATTCAAGCCCTGGAATGAGGCCAGATG<br>TAAGCTCTCCTCCATCTAGCTCCTCAACAACAACGGGACCACCTCCCAAACTCTGCC<br>TGGTGTGCTCCGATGAAGCCTCAGGATGTCATTATGGGGTCTTAACATGTGGAAGCT<br>GTAAAGTGTTCTTCAAAAGAGCAGTGGAAGGACAGCACAATTATCTTTGTGCTGGAA<br>GAAATGATTGTATCATTGATAAAATTCGAAGGAAAAACTGCCCAGCCTGTCGCTATC<br>GGAAATGTCTTCAAGCTGGAATGAACCTAGAAGCTCGAAAAACAAAGAAAAAAATA<br>AAAGGAATTCAACAAACTACGACAGGAGTCTCACAGGATACTTCAGAAAATCTTAA<br>CAAAACAGTAGTTCCTGCAACATTGCCACAACTCACCCCCACCCTGGTGTCTCTCTTG<br>GAGGTCATTGAACCTGAGGTATTATATGCAGGCTATGACTCGTCTGTTCCAGACTCA<br>ACCTGGAGGATCATGACCACACTCAACATGTTAGGTGGGCGCCAAGTGATCGCAGC<br>AGTGAAATGGGCAAAGGCAATACCAGGATTTAAGAACTTACACCTTGATGACCAAA<br>TGATCCTTCTACAGTACTCATGGATGTTTCTAATGGCATTTGCCCTGGGCTGGAGATC<br>ATACAAACAATCAAGTGGAAATTTGCTGTGTTTTGCTCCTGATCTGGTTGTTAATGAG<br>CAGAGAATGACTCTACCCTCCATGTACGAACATTGTAAACATATGCTGTATGTTTCCT<br>CTGAGTTAAAAAGGCTTCAAGTATCTTATGAAGAGTATCTCTGCATGAAAACCTTAC<br>TTCTTCTCTCTTCAGTTCCTAAGGAAGGCTTGAAGAGCCAGGAACTATTTGATGAAA<br>TTAGAATGACCTACATCAAAGAGCTAGGAAAAGCCATTGTCAAAAGGGAAGGAAAC<br>TCCAGCCAGAACTGGCAGAGGTTTTATCAACTGACAAAACTTTTGGATTCGATGCAT<br>GAAGTGGTTGAAAACCTCCTTAGCTATTGCTTCCAAACGTTTTTGGATAAGACCTTG<br>AGTATTGAATTCCCTGAGATGTTAGCTGAAATCATCACCAATCAACTACCAAAATAT<br>TCAAATGGAAATATCAAAAAACTTTTGTTTCATCAAAAATGA
**Mineralocorticoid receptor (MR)**	ATGGAGACCAAAGGCTACCCCAGTCTCCCTGAAGGTCTAGATATGGAAAGGCGGTG<br>GAGTCAAGTTTCTCAGGCTCCAGAAAATTCTTCCCTGGGCGCTACAGAGAGGACCGA<br>TGAGAATAACTACATGGAGATTGTCAACGTAAGCTGTGTTTCCGGTGCTATTCCAAA<br>CAACAGTACTCAAGGAAGCAGCAAAGAAAAACATGAACTACTCCCTTGCCTTCAGC<br>AAGACAATAATCGGTCTGGGATTTTAACATCTGACATTAAAACTGAGCTGGAATCGA<br>AGGAACTTTCAGCTACTGTCGCGGAGTCCATGGGTTTATACATGGATTCTGTAAGAG<br>ACGCTGACTACACATACGATCAGCAGCACCCGCAAGGAAGCCTGAGTCCAGCAAAG<br>ATTTATCAGAATGTAGAGCAGTTGGTGAAATTCTACAAAGAAAATGGCCACCGTCCT<br>TCCACGCTCAGTACTGTGAACAGGCCTTTGAGGCCCTTCGTGTCTGACTCCGGGAGC<br>TCCATGAATGGTGGGGTCTTGCGTGCCATTGTTAAGAGCCCTATCATGTGTCATGAG<br>AAGAGTCCTTCTGTGTGTAGCCCTCTGAACATGACCTCTTCTGTCTGCAGCCCTGCTG<br>GAATCAACTCTGTGTCCTCCACCACAGCTAGCTTTGGCAGTTTCCCGGTGCACAGCC<br>CCATCACCCAAGGAACTCCCCTGACGTGCTCGCCTAACATTGAGAACCGAGGGTCCC<br>GCTCCCACAGCCCTACACATGCTAGCAATGTGGGGTCCCCTCTCTCAAGTCCCTTAG<br>GTAGCATGAAATCCCCAATTTCCAGCCCTCCAAGTCACTGCAGTGTAAAATCTCCGG<br>TCTCCAGTCCTAACGTCACCCTGCGCTCCTCTGTGTCCAGCCCTGCAAATCTCAACAA<br>CTCAAGGTGCTCTGTTTCCAGCCCTTCCAACACCAACAACAGATCCACGCTTTCCAGT<br>CCGACAGCCAGTACCGTGGGATCCATCTGCAGCCCTATAAACAATGCCTTCAGCTAC<br>ACTGCTTCCGGCAACTGTACTGGAGCTGGTGCCCCCCCGGGATGCAATTCCTAGTCCA<br>GACACACATGAGAAAGGTGCTCACGACGTCCCTTTTCCTAAGACTGAGGAAGTCGA<br>GAATGCCATCTCCAATGGTGTGACTAGTCAGCTCAACATTGTCCAGTACATCAAACC<br>AGAACCAGACAGAGCTTTTAGCAGCTCCTGTCTGGGAGGAAACAGCAAAATAAATT<br>CAGACTCTCCATTCTCAGTACCAATAAAGCAAGAACCAACGAAGCATTCGTGTTCGG<br>GCACCTCTTTTAAAGGGAATCCGGCGGTAAACCCGTTTCCATTTATGGACGGCTCAT<br>ATTTTTCCTTTATGGATGATAAAGACTATTATTCTCTATCAGGAATTTTAGGACCACC<br>TGTGCCGGGCTTTGAGGGTAACTGTGAAAGCAGTGCATTCCCAATGGGGATTAAACA<br>AGAACCGGATGATGGGAGCTATTACCCGGAGGCTAGCATCCCTGCCTCTGCCATTGT<br>TGGCGTGAATTCAGGTGGGCAGTCCTTTCACTACAGGATTGGTGCTCAAGGTACAAT<br>ATCTTTATCACGATCAACCAGAGACCAATCTTTCCAGCACCTGAGCTCCTTTCCACCC<br>GTCAGTACTTTAGTGGAGTCCTGGAAATCACATGGCGATTTGTCCTCTAGAAGAAGT<br>GATGGATACCCAGTCCTAGAATACATTCCAGAAAATGTATCAAGCTCTACTTTACGA<br>AGTGTTTCTACTGGATCCTCAAGACCTTCCAAAATATGTTTGGTGTGTGGGGATGAG<br>GCTTCAGGATGTCACTATGGGGTGGTGACCTGTGGCAGCTGCAAAGTCTTCTTCAAA<br>AGAGCAGTGGAAGGGCAACACAATTATTTATGTGCTGGAAGAAATGACTGCATCAT<br>TGATAAGATTCGACGAAAGAACTGTCCAGCCTGCCGACTTCAGAAATGTCTTCAAGC<br>TGGAATGAACTTAGGAGCCCGGAAGTCCAAGAAGCTGGGGAAATTAAAAGGCCTGC<br>ACGAGGAGCACCCCCAGCAGCCGCCGCCTCCCCCTCCGCCCCCGCAGAGCCCCGAG<br>GAAGGGACCACGTACATCGCCCCCACAAAGGAGCCCTCAGTCAACACCGCACTGGT<br>CCCTCAGCTGTCCGCCATCTCTCGGGCCCTCACCCCGTCGCCTGCGATGGTCCTGGAG<br>AGCATCGAGCCGGAAATTGTGTACGCGGGCTATGACAGCTCCAAGCCCGACACCGC<br>GGAAAACCTGCTCTCCACGCTCAACCGCTTGGCCGGCAAGCAGATGATTCAAGTTGT<br>GAAGTGGGCAAAGGTACTTCCAGGATTTAAGAACTTGCCTCTTGAGGACCAAATTAC<br>CCTAATCCAGTATTCTTGGATGTGTCTATCATCATTTGCCTTGAGCTGGAGATCGTAC<br>AAACATACGAACAGCCAATTTCTCTATTTTGCACCAGACCTAGTCTTTAATGAAGAG<br>AAAATGCATCAGTCTGCTATGTATGAACTGTGCCAGGGGATGCATCAGATCAGCCTT<br>CAGTTTGTGCGGCTACAGCTCACCTTTGAAGAATACAGCATAATGAAAGTGCTGCTT

	CTCCTGAGCACAGTTCCAAAGGATGGCCTCAAAAGCCAAGCTGCATTTGAAGAAAT<br>GAGGACAAACTACATCAAAGAACTGAGGAAGATGGTCACCAAGTGTCCCAATAATT<br>CTGGCCAGAGCTGGCAGAGGTTCTATCAGCTGACCAAGCTTCTAGACTCCATGCATG<br>ATCTGGTGAGTGACTTGCTGGAGTTTTGCTTCTATACCTTCCGAGAGTCCCAGGCCCT<br>GAAAGTGGAGTTCCCTGCGATGCTGGTGGAGATCATCAGCGATCAGCTGCCAAAGG<br>TGGAATCTGGCAATGCCAAGGCCCTTTACTTTCACAGGAAGTGA
**Glycogen synthase 1 (Gys1)**	ATGCCTCTGAGCCGGACGTTGTCTGTGTCCTCACTGCCAGGACTGGAGGAGTGGGAG<br>GATGAATTCGATCTGGAGAACACAGTGCTTTTCGAGGTGGCCTGGGAGGTGGCCAAC<br>AAGGTTGGGGGCATCTACACCGTGCTGCAGACCAAGGCGAAGGTGACAGGGGATGA<br>GTGGGGTGACAACTACTACCTGGTGGGACCATACACGGAGCAGGGTGTGAGGACCC<br>AGGTGGAGCTGCTTGAGCCTCCAACCCCGGCCCTGAAGAAGACGCTGGACTCCATG<br>AACAGCAAGGGCTGCAAGGTATATTTCGGGCGCTGGCTGATTGAGGGGGGCCCTCT<br>GGTGGTGCTACTGGATGTGGGGGCCTCTGCCTGGGCCCTGGAACGCTGGAAAGGAG<br>AGCTCTGGGACACCTGCAACATCGGGGTACCCTGGTACGACCGAGAAGCCAATGAC<br>GCTGTCCTCTTTGGCTTCCTCACCACCTGGTTCCTCGGGGAGTTCCTGGCCCAAAGTG<br>AAGAGAAGCCACATGTGGTCGCCCACTTCCACGAGTGGTTGGCAGGTGTGGGGCTCT<br>GCCTATGCCGGGCCCGGCGGCTGCCCGTGGCCACCATCTTCACCACCCATGCCACTC<br>TGCTGGGACGTTACCTGTGTGCTGGAGCTGTGGACTTCTACAACAACTTGGAGAATT<br>TCAATGTAGACAAGGAAGCCGGGGAGAGACAGATCTATCACCGCTACTGCATGGAG<br>CGGGCAGCAGCCCACTGTGCTCACGTCTTCACCACCGTGTCCCAGATAACAGCCATT<br>GAGGCTCAGCACCTGCTCAAGAGGAAGCCAGATGTAATTACCCCAAATGGCTTGAA<br>CGTTAAGAAGTTTTCAGCAGTGCATGAGTTTCAAAATCTGCATGCCATGTACAAGGC<br>CAGGATCCAAGATTTTGTTCGAGGTCATTTCTATGGTCATCTTGACTTCGATCTAGAA<br>AAGACTTTATTCCTTTTCATTGCGGGGAGGTATGAGTTTTCCAACAAAGGAGCAGAC<br>ATCTTCCTAGAATCTTTATCCAGACTAAACTTCTTGCTGAGGATGCATAAAAGTAAC<br>GTCACTGTGGTGGTGTTTTTCATCATGCCTGCCAAGACAAACAATTTCAACGTAGAA<br>ACATTAAAAGGACAGGCTGTGCGGAAGCAGCTATGGGACACTGCACATTCAATGAA<br>GGAAAAGTTTGGAAAGAAACTCTATGATGGATTGTTAAGAGGAGAAATTCCTGACA<br>TGAATAATATTTTGGATCGAGATGACCTAACAATTATGAAAAGAGCCATTTTTTCAA<br>CTCAGCGACACTCTCTGCCACCCGTGACAACTCACAACATGATCGAGGACTCCACAG<br>ATCCCATCCTCAGCACCATTAGACGGCTCGGGCTTTTCAACAGCCGCTCAGACAGAG<br>TCAAGGTAGGAATCATTTTGCACCCAGAGTTCCTATCCTCCACTAGCCCTCTGTTGCC<br>CATGGATTATGAAGAGTTCGTCCGAGGTTGTCATCTTGGTGTGTTCCCATCATACTAT<br>GAACCCTGGGGCTATACTCCAGCTGAGTGTACCGTCATGGGCATCCCCAGTATCTCC<br>ACAAACCTCTCTGGCTTCGGCTGCTTCATGGAGGAGCACATTGCAGACCCCTCTGCT<br>TACGGCATCTACATTCTGGATCGGCGGTTCCGCAGCCTGGACGATTCCTGTTCACAG<br>CTCACCTCCTTCCTCTACAGCTTCTGCCAGCAGAGCCGCAGACAGCGCATCATCCAG<br>CGGAACCGCACAGAGCGTCTCTCCGACCTTCTGGACTGGAAGTACCTAGGCCGATAT<br>TACATGTCTGCGCGCCACATGGCATTGGCCAAGGCCTTTCCAGATCACTTCACGTAT<br>GAGCCCCACGAGACCGATGCGACCCAGGGCTACCGCTACCCAAGGCCAGCCTCGGT<br>GCCACCCTCGCCCTCACTGTCGCGGCACTCAAGCCCACACCAGAGCGAGGATGAGG<br>AGGAACCCCGGGAAGGGCCCCTCGAGGAAGATGGCGAACGATATGACGAGGACGA<br>AGAGGCTGCTAAGGATCGGCGCAACATCCGCGCGCCCGAGTGGCCACGCCGCGCCT<br>CCTGCACCCCTTCCCCCGGCAAGCGCAGCAACTCAGTGGACACTGCGCCCTCCAGCT<br>CTCTGAGCACACCGAGTGAGCCCCTCAGCCCCGCTAGCTCCCTGGGGGAGGAGCGC<br>AACTAA

Table 1C. List of *Rattus norvegicus* protein and mRNA sequence IDs used to search for its *Myocastor coypus* ortholog

Gene Name	Gene Symbol	Rat RefSeq mRNA ID	Rat RefSeq protein ID
Gonadotropin-releasing hormone	GNRH	NM_031038.3	NP_112300.2
Gonadotropin-releasing hormone receptor	GNRHR	XM_005373712.2*	XP_005373769.1*
Androgen receptor	AR	NM_012502.1	NP_036634.1

Aromatase	CYP19A1, aromatase	NM_017085.2	NP_058781.2
Estrogen receptor α	ESR1	NM_012689.1	NP_036821.1
Glucocorticoid receptor	Nr3c1, GR	NM_012576.2	NP_036708.2
Mineralocorticoid receptor	Nr3c2, MR	NM_013131.1	NP_037263.1

Table 2: Protein sequences used for analysis of homology

**Organism**	**Protein**	***R. norvegicus***	***M. musculus***	***H. sapiens***
M. coypus	GNRH	NP_036899.1	NP_032171.1	NP_000816.4
	GNRHR	NP_112300.2	NP_034453.1	NP_000397.1
	AR	NP_036634.2	NP_038504.1	AA51770.1
	aromatase	NP_058781.2	NP_001335102.1	NP_001334185.1
	ESR1	NP_036821.1	NP_001289461.1	NP_001372497.1
	ESR2	NP_036886.3	NP_997590.1	NP_001428.1
	Nr3c1	NP_036708.2	NP_001348138.1	NP_001351110.1
	Nr3c2	NP_001382006.1	NP_001077375.1	NP_000892.2
	Srda5a1	NP_058766.2	NP_780492.2	NP_001038.1
	Srda5a2	NP_073202.1	NP_444418.1	NP_000339.2

Table 3: HPG axis gene expression in the prefrontal cortex. Maternal ID and year were included in each model as random effects. Hair T is the level of testosterone in fetal hair samples. P - values below 0.05 are in **bold**. DF = 1 for all model effects.

Gene	Model Effects	Log Worth	Estimate	F Ratio	P-Value
GnRH (n = 24)	Sex<br>IUP<br>Sex*IUP<br>Hair T	0.657<br>0.943<br>0.786<br>1.257	0.197<br>0.373<br>0.201<br>0.570	1.632<br>7.450<br>2.123<br>4.198	0.220<br>0.114<br>0.164<br>0.055
GNRHR (n = 20)	**Sex**<br>IUP<br>Sex*IUP<br>Hair T	**1.449**<br>0.510<br>0.713<br>1.200	**0.186**<br>0.106<br>0.117<br>0.321	**5.409**<br>1.111<br>1.858<br>4.054	**0.036**<br>0.309<br>0.193<br>0.063
AR (n = 24)	Sex<br>IUP<br>Sex*IUP<br>Hair T	0.871<br>0.727<br>0.550<br>0.792	0.164<br>0.183<br>-0.112<br>0.255	2.454<br>1.871<br>1.239<br>2.128	0.135<br>0.187<br>0.282<br>0.161
aromatase (n = 23)	Sex<br>IUP<br>Sex*IUP<br>Hair T	0.860<br>0.582<br>1.094<br>0.898	0.144<br>0.124<br>-0.167<br>0.316	2.453<br>1.365<br>3.502<br>2.607	0.138<br>0.262<br>0.081<br>0.127
ESR1 (n = 24)	Sex<br>IUP<br>Sex*IUP<br>Hair T	0.178<br>0.048<br>0.038<br>0.152	0.100<br>0.112<br>-0.063<br>0.328	0.195<br>0.028<br>0.017<br>0.184	0.664<br>0.895<br>0.916<br>0.704

Table 4: HPG axis gene expression in the hypothalamus. Maternal ID and year were included in each model as random effects. AF T is the level of testosterone in fetal AF samples. P - values below 0.05 are in **bold**. DF = 1 for all model effects.

Gene	Model Effects	Log Worth	Estimate	F Ratio	P-Value
GnRH (n = 20)	Sex	0.508	-0.181	1.194	0.310
	**IUP**	**2.492**	**-0.627**	**17.553**	**0.003**
	Sex*IUP	0.473	0.178	1.049	0.337
	AF T	1.194	-0.072	4.239	0.064
GNRHR (n = 18)	Sex	1.022	-0.363	3.252	0.095
	IUP	0.081	-0.046	0.048	0.830
	**Sex*IUP**	**1.956**	**-0.433**	**9.034**	**0.011**
	AF T	0.658	-0.118	5.546	0.220
AR (n = 22)	Sex	0.118	0.026	0.095	0.762
	IUP	0.094	0.018	0.062	0.806
	Sex*IUP	0.101	-0.023	0.071	0.793
	AF T	0.238	0.013	0.341	0.578
aromatase (n = 20)	Sex	0.384	0.145	0.723	0.413
	IUP	0.701	-0.213	1.868	0.199
	Sex*IUP	0.446	-0.174	0.916	0.358
	AF T	0.454	0.038	0.928	0.352
ESR1 (n = 22)	Sex	0.967	0.197	2.912	0.108
	IUP	0.613	-0.126	1.476	0.244
	Sex*IUP	0.055	-0.018	0.023	0.881
	AF T	0.111	-0.008	0.089	0.774

Table 5: HPG axis gene expression in the prefrontal cortex. Maternal ID and year were included in each model as random effects. AF T is the level of testosterone in fetal AF samples. P - values below 0.05 are in **bold**. DF = 1 for all model effects.

**Gene**	**Model Effects**	**Log Worth**	**Estimate**	**F Ratio**	**P-Value**
GnRH (n = 21)	Sex	0.198	-0.084	0.237	0.634
	IUP	0.487	-0.180	1.040	0.326
	Sex*IUP	0.097	0.050	0.067	0.800
	AF T	0.414	0.030	0.807	0.385
GNRHR (n = 19)	Sex	0.259	0.063	0.380	0.551
	**IUP**	**1.489**	**-0.231**	**5.943**	**0.032**
	Sex*IUP	0.621	0.129	1.544	0.240
	AF T	0.194	-0.011	0.231	0.639
AR (n = 23)	Sex	0.364	0.081	0.658	0.433
	IUP	0.436	-0.085	0.887	0.366
	Sex*IUP	0.727	0.137	1.945	0.187
	**AF T**	**2.057**	**-0.064**	**10.185**	**0.009**
aromatase (n = 21)	Sex	0.036	-0.012	0.011	0.920
	IUP	0.022	-0.007	0.004	0.950
	Sex*IUP	0.378	-0.098	0.705	0.419
	AF T	0.221	-0.014	0.288	0.601
ESR1 (n = 23)	Sex	0.121	0.076	0.099	0.757
	IUP	0.291	-0.145	0.448	0.512
	Sex*IUP	0.376	0.207	0.680	0.420
	AF T	0.185	-0.043	0.307	0.652

Table 6: HPG axis gene expression in the striatum. Maternal ID and year were included in each model as random effects. AF T is the level of testosterone in fetal AF samples. P - values below 0.05 are in **bold**. DF = 1 for all model effects.

Gene	Model Effects	Log Worth	Estimate	F Ratio	P-Value
GnRH (n = 16)	Sex	0.649	0.272	1.714	0.225
	IUP	0.983	0.363	3.221	0.104
	Sex*IUP	0.073	0.048	0.040	0.846
	AF T	0.003	0.0004	0.0001	0.994
GNRHR (n = 13)	Sex	0.217	0.195	0.287	0.607
	IUP	0.030	0.029	0.008	0.932
	**Sex*IUP**	**1.647**	**-0.638**	**7.941**	**0.023**
	AF T	0.603	-0.076	1.542	0.250
AR (n = 17)	Sex	0.534	0.150	1.215	0.292
	IUP	0.161	0.052	0.167	0.691
	Sex*IUP	0.166	-0.059	0.176	0.683
	AF T	0.000	-2.431e-5	0.000	0.999
aromatase (n = 16)	Sex	1.157	0.571	4.102	0.070
	IUP	0.304	0.175	0.496	0.497
	Sex*IUP	0.177	-0.088	0.199	0.665
	AF T	0.919	-0.079	2.849	0.120
ESR1 (n = 16)	Sex	1.042	0.237	3.519	0.091
	IUP	0.723	0.187	1.973	0.189
	Sex*IUP	0.598	0.178	1.458	0.253
	AF T	0.205	0.014	0.261	0.624

Table 7: HPG axis gene expression in the striatum. Maternal ID and year were included in each model as random effects. Hair T is the level of testosterone in fetal hair samples. P - values below 0.05 are in **bold**. DF = 1 for all model effects.

Gene	Model Effects	Log Worth	Estimate	F Ratio	P-Value
GnRH (n = 22)	Sex	0.080	0.071	0.047	0.831
	IUP	0.122	-0.118	0.100	0.756
	Sex*IUP	0.480	0.322	1.003	0.331
	Hair T	0.362	-0.464	0.642	0.434
GNRHR (n = 16)	Sex	0.277	-0.229	0.431	0.528
	IUP	0.475	0.386	1.040	0.335
	Sex*IUP	0.184	0.197	0.212	0.655
	Hair T	0.571	0.678	1.392	0.269
AR (n = 22)	Sex	0.354	0.087	0.623	0.443
	IUP	0.724	0.166	1.902	0.189
	**Sex*IUP**	**1.363**	**0.231**	**4.907**	**0.043**
	Hair T	0.251	-0.113	0.354	0.561
aromatase (n = 22)	Sex	0.284	0.411	0.432	0.521
	IUP	0.492	-0.730	1.046	0.322
	Sex*IUP	0.050	0.115	0.020	0.892
	Hair T	0.327	-0.937	0.549	0.471
ESR1 (n = 22)	Sex	0.023	-0.009	0.004	0.948
	IUP	0.458	0.150	0.935	0.348
	Sex*IUP	0.567	0.157	1.294	0.271
	Hair T	0.649	-0.306	1.589	0.225

Table 8: HPG axis gene expression in the hypothalamus. Maternal ID and year were included in each model as random effects. Hair T is the level of testosterone in fetal hair samples. P - values below 0.05 are in **bold**. DF = 1 for all model effects.

Gene	Model Effects	Log Worth	Estimate	F Ratio	P-Value
GnRH (n = 23)	Sex	0.014	0.008	0.002	0.969
	**IUP**	**1.674**	**-0.527**	**6.592**	**0.021**
	Sex*IUP	0.540	-0.202	1.210	0.288
	Hair T	0.037	0.037	0.011	0.918
GNRHR (n = 21)	Sex	0.037	-0.040	0.011	0.918
	IUP	0.030	-0.033	0.007	0.94
	Sex*IUP	0.028	-0.038	0.009	0.938
	Hair T	0.042	0.082	0.014	0.908
AR (n = 23)	Sex	0.010	0.002	0.001	0.978
	IUP	0.178	0.036	0.197	0.663
	Sex*IUP	0.431	-0.066	0.849	0.371
	Hair T	0.132	0.046	0.116	0.738
aromatase (n = 23)	Sex	0.101	0.049	0.072	0.792
	IUP	0.831	-0.316	2.310	0.148
	Sex*IUP	1.032	-0.318	3.188	0.093
	Hair T	0.007	-0.007	0.0004	0.985
ESR1 (n = 23)	Sex	1.130	0.168	3.621	0.074
	IUP	1.263	-0.196	4.246	0.055
	**Sex*IUP**	**2.773**	**-0.209**	**13.611**	**0.002**
	Hair T	0.104	-0.040	0.075	0.787

Table 9: HPA axis gene expression in the hypothalamus. Maternal ID and year were included in each model as random effects. AF cortisol is the level of cortisol in fetal AF samples. P - values below 0.05 are in **bold**. DF = 1 for all model effects.

Gene	Model Effects	Log Worth	Estimate	F Ratio	P-Value
GR (n = 34)	**Sex**	**1.843**	**0.185**	**6.982**	**0.014**
	IUP	0.513	0.076	1.091	0.307
	Sex*IUP	0.729	0.096	1.848	0.187
	AF cortisol	0.111	-0.0001	0.085	0.774
MR (n = 34)	Sex	1.144	0.107	3.553	0.072
	IUP	0.145	0.022	0.136	0.715
	Sex*IUP	0.263	0.035	0.376	0.545
	AF cortisol	0.087	-8.85e-5	0.054	0.818

Table 10: HPA axis gene expression in the prefrontal cortex. Maternal ID and year were included in each model as random effects. Hair cortisol is the level of cortisol in fetal hair samples. P - values below 0.05 are in **bold**. DF = 1 for all model effects.

Gene	Model Effects	Log Worth	Estimate	F Ratio	P-Value
GR (n = 23)	Sex	0.109	-0.013	0.082	0.779
	IUP	0.032	-0.005	0.008	0.929
	Sex*IUP	0.685	0.066	1.735	0.206
	Hair cortisol	0.914	-0.005	2.636	0.122
MR (n = 23)	Sex	0.411	-0.05	0.79	0.388
	IUP	1.097	0.13	3.510	0.08
	**Sex*IUP**	**1.852**	**0.164**	**7.611**	**0.014**
	**Hair cortisol**	**1.685**	**-0.009**	**6.464**	**0.021**

Table 11: HPA axis gene expression in the prefrontal cortex. Maternal ID and year were included in each model as random effects. AF cortisol is the level of cortisol in fetal AF samples. P - values below 0.05 are in **bold**. DF = 1 for all model effects.

Gene	Model Effects	Log Worth	Estimate	F Ratio	P-Value
GR<br>(n = 35)	Sex<br>IUP<br>Sex*IUP<br>AF cortisol	0.195<br>0.173<br>0.453<br>0.497	0.030<br>-0.028<br>0.061<br>-0.0004	0.227<br>0.185<br>0.899<br>1.040	0.638<br>0.671<br>0.352<br>0.318
MR<br>(n = 35)	Sex<br>IUP<br>Sex*IUP<br>AF cortisol	0.093<br>0.046<br>0.603<br>0.216	0.023<br>0.011<br>0.097<br>-0.0006	0.063<br>0.017<br>1.50<br>0.396	0.806<br>0.899<br>0.249<br>0.607

Table 12: HPA axis gene expression in the striatum. Maternal ID and year were included in each model as random effects. AF cortisol is the level of cortisol in fetal AF samples. P - values below 0.05 are in **bold**. DF = 1 for all model effects.

Gene	Model Effects	Log Worth	Estimate	F Ratio	P-Value
GR<br>(n = 29)	Sex<br>IUP<br>Sex*IUP<br>AF cortisol	0.172<br>0.229<br>0.538<br>0.063	0.037<br>-0.052<br>-0.096<br>-6.76e-5	0.183<br>0.299<br>1.181<br>0.031	0.674<br>0.591<br>0.290<br>0.864
MR<br>(n = 29)	Sex<br>IUP<br>Sex*IUP<br>AF cortisol	0.818<br>0.003<br>0.091<br>0.011	0.243<br>-0.001<br>-0.039<br>2.19e-5	2.219<br>0.0001<br>0.059<br>0.001	0.152<br>0.994<br>0.811<br>0.976

Table 13: HPA axis gene expression in the hypothalamus. Maternal ID and year were included in each model as random effects. Hair cortisol is the level of cortisol in fetal hair samples. P - values below 0.05 are in **bold**. DF = 1 for all model effects.

Gene	Model Effects	Log Worth	Estimate	F Ratio	P-Value
GR (n = 22)	Sex	0.799	0.102	2.191	0.159
	IUP	0.004	-0.001	0.0001	0.992
	Sex*IUP	0.713	-0.101	1.841	0.194
	Hair cortisol	0.010	0.0001	0.001	0.977
MR (n = 22)	Sex	0.188	0.026	0.215	0.649
	IUP	0.210	-0.035	0.261	0.616
	Sex*IUP	0.786	-0.079	2.132	0.164
	Hair cortisol	0.829	0.002	2.306	0.148

Table 14: HPA axis gene expression in the striatum. Maternal ID and year were included in each model as random effects. Hair cortisol is the level of cortisol in fetal hair samples. P - values below 0.05 are in **bold**. DF = 1 for all model effects.

Gene	Model Effects	Log Worth	Estimate	F Ratio	P-Value
GR (n = 21)	Sex	0.369	0.045	0.663	0.427
	IUP	0.020	-0.003	0.003	0.955
	Sex*IUP	0.125	0.017	0.105	0.751
	**Hair cortisol**	**3.533**	**-0.007**	**21.199**	**0.0003**
MR (n = 21)	Sex	0.189	0.183	0.23	0.648
	IUP	0.180	0.139	0.217	0.661
	Sex*IUP	0.065	0.040	0.035	0.861
	Hair cortisol	0.009	0.001	0.001	0.98

Table 15. List of housekeeping genes with their *Rattus norvegicus* protein and mRNA sequence IDs used to search for its *Myocastor coypus* ortholog

Gene	Rat RefSeq AA ID	Rat RefSeq mRNA ID
Glycogen [starch] synthase (Gys1)	NM_001109615.1	NP_001103085.1

Table 16. *Myocastor coypus* primers for qPCR

Gene	Product Size	Forward/Reverse Primers
Androgen receptor (AR)	114	F: 5`-GGACCCTGGATGGAGAGCTA-3`<br>R: 5`-GATCAGGCAGGTCTTCTGGG-3`
Gonadotropin-releasing hormone (GNRH)	70	F: 5`-ACCCTGGTTGAGTCTTTCCA-3`<br>R: 5`-CGAGATGCTGCTGGGGTT-3`
Gonadotropin-releasing hormone receptor (GNRHR)	90	F: 5`-TCCTCAGTAGTGCCTTTGCTG-3`<br>R: 5`-GGGAGAAAACTTCTGTTTGTCCAG-3`
Estrogen receptor α (ESR1)	81	F: 5`-CCTTCTACAGGCCAAATTCAGG-3`<br>R: 5`-CTATGCTTCCCTTGTCGCTG-3`
Aromatase	83	F: 5`-TCCTGCTGCTTATGGGCTTC-3`<br>R: 5`-TCCCAGACAGTAGCCAGGAC-3`
Glucocorticoid receptor (GR)	87	F: 5`-AAGAGCAGTGGAAGGACAGC-3`<br>R: 5`-GCTGGGCAGTTTTTCCTTCG-3`
Mineralocorticoid receptor (MR)	131	F: 5`-ACTATGGGGTGGTGACCTGT-3`<br>R: 5`-GGCTGGACAGTTCTTTCGTC-3`
Glycogen synthase 1 (Gys1)	170	F: 5`-GACGTTGTCTGTGTCCTCACT-3`<br>R: 5`-AGTTGTCACCCCACTCATCC-3`

Table 17: Mass spectrometer conditions

Parameter	Value
Curtain gas	35 psi
Temperature	600 ˚C
Ion Source Gas 1	35 psi
Ion Source Gas 2	60 psi
Collision Gas	Medium
Nebulizer Current	5 µA

Table 18: MRM conditions for the steroids analyzed in APCI positive mode. (DP= declustering potential, EP= entrance potential, CE= collision energy, and CXP= collision cell exit potential).

Analyte & Internal STD	MRM Transitions	DP (V)	EP (V)	CE (eV)	CXP (V)
Cortisol	363/121<br>363/327	80	10	32	12
Cortisol-d4	367/121<br>367/331	80	10	32	12
Testosterone	289/91<br>289/109	80	10	31	12
Testosterone-d2	291/99<br>291/111	80	10	31	12

Table 19: Concentrations (ng/mL) of calibrators prepared in Methanol:H2O (50/50, v/v). R^2 of the calibration curve was > 0.99.

Compound	STD 1	STD 2	STD 3	STD 4	STD 5	STD 6	STD 7	STD 8	STD 9	STD 10
cortisol	250	100	50	25	10	5	2.5	1	0.5	0.25
testosterone	25	10	5	2.5	1	0.5	0.25	0.1	0.05	0.025

Table 20: AICc values in the hypothalamus for HPG components. The lowest values are in bold and show the parameter used in the subsequent models.

Gene	Estimated gestational age	Fetal weight	Residual fetal weight & estimated gestational age
GnRH	**222.53**	226.46	226.12
GNRHR	**189.24**	192.7	189.96
AR	**90.24**	99.91	92.95
aromatase	**199.79**	202.97	200.64
ESR1	124.36	128.44	**122.96**

Table 21: AICc values in the cortex for HPG components. The lowest values are in bold and show the parameter used in the subsequent models.

Gene	Estimated gestational age	Fetal weight	Residual fetal weight & estimated gestational age
GnRH	**183.77**	187.52	187.7
GNRHR	**126.04**	128.47	128.96
AR	**153.68**	157.01	157.15
aromatase	**154.38**	154.6	165
ESR1	**192.14**	195.86	199.07

Table 22: AICc values in the striatum for HPG components. The lowest values are in bold and show the parameter used in the subsequent models.

Gene	Estimated gestational age	Fetal weight	Residual fetal weight & estimated gestational age
GnRH	**160.59**	201.57	168.69
GNRHR	**154.09**	157.56	156.77
AR	**101.54**	105.95	103.85
aromatase	**192.87**	195.96	195.46
ESR1	**104.16**	106.82	107.09

Table 23: AICc values in the hypothalamus for HPA components. The lowest values are in bold and show the parameter used in the subsequent models.

Gene	SBL	Estimated gestational age	Fetal weight	Residual fetal weight & estimated gestational age	Residual SBL & estimated gestational age
GR	**91.93**	105.87	112.78	112.35	94.73
MR	**73.56**	81.18	90.62	87.25	74.8

Table 24: AICc values in the cortex for HPA components. The lowest values are in bold and show the parameter used in the subsequent models.

**Gene**	**SBL**	**Estimated gestational age**	**Fetal weight**	**Residual fetal weight & estimated gestational age**	**Residual SBL & estimated gestational age**
GR	94.55	98.98	102.63	102.67	**94.22**
MR	91.04	100.56	103.86	104.55	**90.92**

Table 25: AICc values in the striatum for HPA components. The lowest values are in bold and show the parameter used in the subsequent models.

**Gene**	**SBL**	**Estimated gestational age**	**Fetal weight**	**Residual fetal weight & estimated gestational age**	**Residual SBL & estimated gestational age**
GR	88.57	97.35	101.84	101.1	**88.42**
MR	129.75	152.7	158.46	155.63	**128.8**
